# 17^th^ Century Variola Virus Reveals the Recent History of Smallpox

**DOI:** 10.1016/j.cub.2016.10.061

**Published:** 2016-12-19

**Authors:** Ana T. Duggan, Maria F. Perdomo, Dario Piombino-Mascali, Stephanie Marciniak, Debi Poinar, Matthew V. Emery, Jan P. Buchmann, Sebastian Duchêne, Rimantas Jankauskas, Margaret Humphreys, G. Brian Golding, John Southon, Alison Devault, Jean-Marie Rouillard, Jason W. Sahl, Olivier Dutour, Klaus Hedman, Antti Sajantila, Geoffrey L. Smith, Edward C. Holmes, Hendrik N. Poinar

**Affiliations:** 1 McMaster Ancient DNA Centre, Department of Anthropology, McMaster University, Hamilton, ON L8S 4L8, Canada; 2 Department of Virology, University of Helsinki, Helsinki 00014, Finland; 3 Department of Anatomy, Histology, and Anthropology, Faculty of Medicine, Vilnius University, Vilnius 03101, Lithuania; 4 Marie Bashir Institute for Infectious Diseases and Biosecurity, Charles Perkins Centre, School of Life and Environmental Sciences and Sydney Medical School, The University of Sydney, Sydney, NSW 2145, Australia; 5 Department of Biochemistry and Molecular Biology, Bio21 Molecular Science and Biotechnology Institute, The University of Melbourne, Melbourne, VIC 3010, Australia; 6 Department of History, Duke University, Durham, NC 27708-0719, USA; 7 Department of Biology, McMaster University, Hamilton, ON L8S 4L8, Canada; 8 Keck Carbon Cycle Accelerator Mass Spectrometer, Earth Systems Science Department, University of California, Irvine, CA 92697-3100, USA; 9 MYcroarray, Ann Arbor, MI 48105, USA; 10 Department of Chemical Engineering, University of Michigan, Ann Arbor, MI 48109-2136, USA; 11 Center for Microbial Genetics and Genomics, Northern Arizona University, Flagstaff, AZ 86011-4073, USA; 12 Laboratoire d’Anthropologie Biologique Paul Broca, Ecole Pratique des Hautes Etudes, PSL Research University, Paris 75014, France; 13 PACEA, CNRS, Université de Bordeaux, Pessac 33615, France; 14 Helsinki University Hospital, Helsinki 00029, Finland; 15 Department of Forensic Medicine, University of Helsinki, Helsinki 00014, Finland; 16 Department of Pathology, University of Cambridge, Cambridge CB2 1QP, UK; 17 Michael G. DeGroote Institute for Infectious Disease Research, McMaster University, Hamilton, ON L8S 4L8, Canada; 18 Humans and the Microbiome Program, Canadian Institute for Advanced Research, Toronto, ON M5G 1Z8, Canada

**Keywords:** variola virus, smallpox, ancient DNA, Lithuanian Mummy Project, evolution, molecular clock, phylogeny

## Abstract

Smallpox holds a unique position in the history of medicine. It was the first disease for which a vaccine was developed and remains the only human disease eradicated by vaccination. Although there have been claims of smallpox in Egypt, India, and China dating back millennia [1, 2, 3, 4], the timescale of emergence of the causative agent, variola virus (VARV), and how it evolved in the context of increasingly widespread immunization, have proven controversial [4, 5, 6, 7, 8, 9]. In particular, some molecular-clock-based studies have suggested that key events in VARV evolution only occurred during the last two centuries [4, 5, 6] and hence in apparent conflict with anecdotal historical reports, although it is difficult to distinguish smallpox from other pustular rashes by description alone. To address these issues, we captured, sequenced, and reconstructed a draft genome of an ancient strain of VARV, sampled from a Lithuanian child mummy dating between 1643 and 1665 and close to the time of several documented European epidemics [1, 2, 10]. When compared to vaccinia virus, this archival strain contained the same pattern of gene degradation as 20^th^ century VARVs, indicating that such loss of gene function had occurred before ca. 1650. Strikingly, the mummy sequence fell basal to all currently sequenced strains of VARV on phylogenetic trees. Molecular-clock analyses revealed a strong clock-like structure and that the timescale of smallpox evolution is more recent than often supposed, with the diversification of major viral lineages only occurring within the 18^th^ and 19^th^ centuries, concomitant with the development of modern vaccination.

## Highlights

• Variola virus genome was reconstructed from a 17^th^ century mummified child
• The archival strain is basal to all 20^th^ century strains, with same gene degradation
• Molecular-clock analyses show that much of variola virus evolution occurred recently

## Teaser

Using ancient DNA sequences of variola virus recovered from the mummified remains of a 17^th^ century child, Duggan et al. reconstruct the evolutionary history of smallpox. With the ancient strain, the genetic diversification of the smallpox virus is found to be more recent than previously supposed and concurrent with the onset of widespread vaccination.

## Results and Discussion

**Figure 1:**
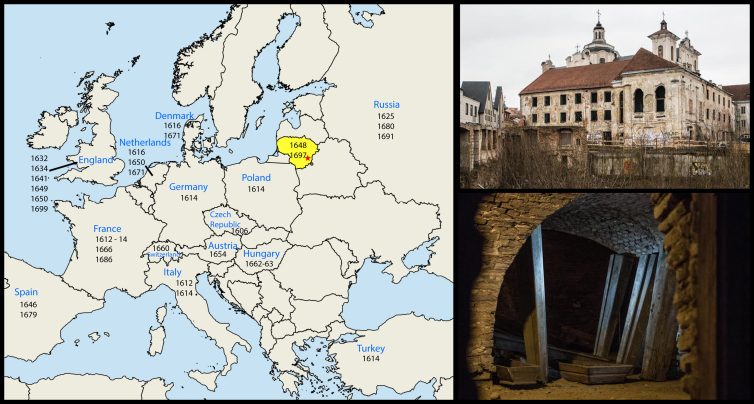
Dominican Church of the Holy Spirit, Vilnius Left: Lithuania is shaded in yellow, with the red star indicating the city of Vilnius, the location of the Dominican Church of the Holy Spirit where the VD21 specimen was found and dated to approximately 1654. Dates in black indicate known smallpox outbreaks in nearby countries during the 17^th^ century [1, 2]. Top right: the Dominican Church of the Holy Spirit in Vilnius, Lithuania. Bottom right: the crypt where the child mummy was located. See also Figure S1 and Table S1.

In an attempt to reveal the evolutionary history of smallpox (variola virus, VARV), we sampled the partial mummified remains of a young child of undetermined sex found within the crypt of the Dominican Church of the Holy Spirit of Vilnius, Lithuania, with no associated artifacts or coffin [11, 12] (Figures 1 and S1). As the bones were covered by soft tissue, it was difficult to obtain a precise age at death, although it is thought to be between 2 and 4 years [13]. Radiocarbon dating of the sample yielded a ^14^C age of 250 ± 15 BP, which at 2 SDs calibrates to 1643–1665 AD (relative probability, p = 0.93) or 1785–1793 (p = 0.07) (Table S1; Figure S2). The older calibrated age range, which contains the bulk of the probability density function, agrees with historical sources that place this during the Russian occupation of 1655–1661 [12], as well as the reported presence of endemic smallpox within Lithuania. Our older date is also supported by a Bayesian molecular-clock analysis, which gave mean sampling time estimates of 1691 and 1665 under constant size and Bayesian skygrid demographic models, respectively (see the Supplemental Experimental Procedures for further details).

**Figure 2:**
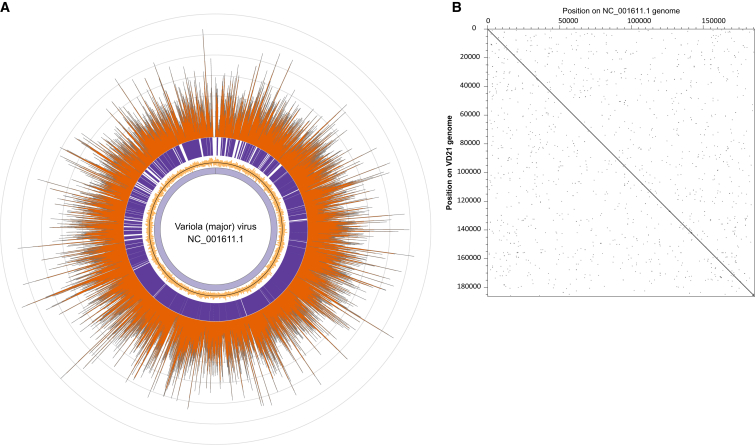
VARV Genome Reconstructed from VD21 (A) Coverage of reference NC_001611.1 variola (major) virus genome. The inner-most circle (light purple) indicates the full 185,578 bp length of the reference, and the inner yellow circle depicts GC content across the reference genome with the genomic average of 32.7% indicated by the thin dark ring. The dark-purple ring indicates the location of annotated genes in the reference. The outer-most ring (dark orange) represents the coverage depth of reads from sample VD21 mapped to the NC_001611.1 reference sequence averaged across 25 bp windows. Average coverage was 18× (minimum 0× to maximum 60×). The concentric gray lines represent intervals of 10× coverage. The plot was constructed with Circos [14]. (B) Conservation of genomic sequence between VD21 and the VARV reference genome NC_001611.1. The plot was constructed with Dotter [15]. See also Figure S2.

BLAST analysis of a library, enriched for an unrelated target (JC polyomavirus), indicated that of 0.03% of the 1.3 million hits assigned to viruses, 47% (198 reads) were top hits to VARV. To confirm the presence of this virus, we enriched the library using a custom in-solution bait set targeting publicly available strains of VARV and sequenced 845,594 reads (Supplemental Experimental Procedures). We were able to map 43,243 reads to a reference strain of variola (major) virus (India 1967; GenBank: NC_001611.1) and from this reconstructed an ancient VARV genome at an average of 18× coverage (range 0×–60×) (Figure 2). Additionally, through de novo assembly, we reconstructed a draft viral genome that is 187,565 bp in length and contains all annotated genes found in the VARV reference sequence (Supplemental Experimental Procedures). We investigated the synteny in our draft genome by aligning it to the variola major virus reference genome sequence [15]. This revealed that the genome of VD21 shows no major rearrangements and a strong conservation in gene content and arrangement with all other VARVs isolated in the 20^th^ century (Figure 2).

Overall, we identified and confirmed a total of 716 nucleotide substitutions relative to the VARV reference sequence by eye and using NASP [16] in conjunction with GATK [17], with a minimum of 5× coverage and 0.9 frequency. We also enriched for, sequenced, and produced a mitochondrial genome at 193× coverage. The mitochondrial haplogroup of VD21 was H2a5b (Supplemental Experimental Procedures). Haplogroup H2 and its descendent lineages are common throughout Europe. The presence of a common European haplogroup, as well as the DNA damage profiles and overall fragment length distribution of the reads mapped to both VARV and the mitochondrial genome, supports the presence of authentic ancient DNA.

VARV genomes are characterized by the fragmentation of several genes, such that they are non-functional, even though their homologs are intact in vaccinia virus (VACV; GenBank: NC_006998.1) and in other orthopoxviruses [18]. Notably, VD21 exhibited the same pattern of gene disruption in comparison to VACV as more modern strains of VARV, indicating that the loss of gene function during VARV evolution had occurred prior to ca. 1650. However, VD21 also carried a number of additional amino acid substitutions and nonsense or frameshift mutations that might alter gene function, although this remains to be determined.

**Figure 3:**
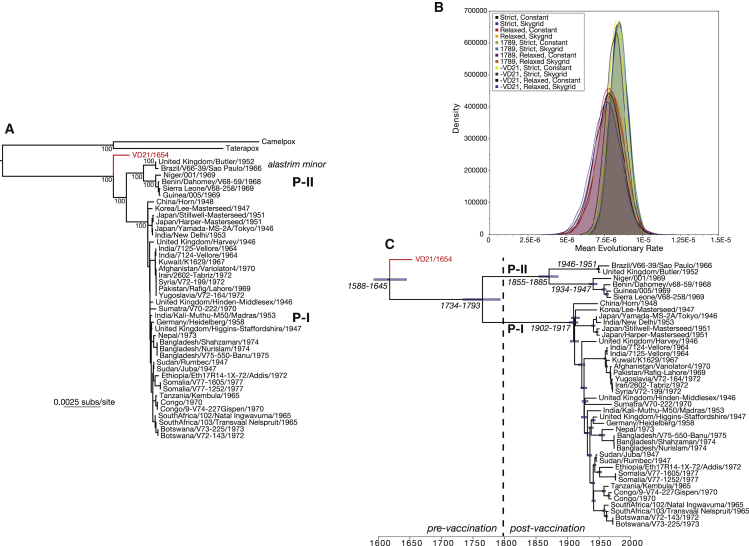
Evolutionary History of VARV (A) Maximum-likelihood phylogeny of 43 strains of VARV rooted using homologous sequences from camels (camelpox) and African gerbils (taterapox), which are the orthopoxviruses most closely related to VARV. All horizontal branch lengths are drawn to a scale of nucleotide substitutions per site, and bootstrap values are shown for key nodes, with year of sampling shown for the VARV strains. The VD21 strain is shown in red, and the major clades of VARV (P-I and P-II, with the later the containing alastrim minor strains) are marked. (B) Posterior probability densities of mean evolutionary rate estimates for VARV under different molecular-clock and coalescent models, possible ^14^C dates for VD21 (i.e., “1789” assumes VD21 is from 1789 rather than 1654), and with VD21 excluded (i.e., “−VD21”) from the analysis (see Table S3 for full results). (C) Maximum clade credibility tree showing the timescale of VARV evolution inferred under a strict molecular clock and a constant population size, with the VD21 lineage shown in red and the 95% credible intervals for a number of other key divergence events also shown. The date (1796) of the introduction of the cowpox-based smallpox vaccine by Jenner, which we include as an historical reference point only, is marked by a hatched line, and it is important to note that a process of smallpox variolation (inoculation) had most likely been in existence for many centuries prior to this. See also Figure S3 and Tables S2 and S3.

Phylogenetic analysis of VD21 with 42 additional complete genomes of VARV (Table S2) and the two most closely related orthopoxviruses (camelpox and taterapox) utilized as outgroups clearly placed our ancient strain basal to all previously sequenced VARV strains (Figure 3). The divergent phylogenetic position of VD21 is seemingly similar to that observed in a partial VARV sequence obtained from a 300-year-old Siberian mummy [19], although the 718 bp sequence obtained from that sample provided little phylogenetic resolution (Figure S3). The basal phylogenetic position of VD21 clearly indicates that the previously described genetic diversity of VARV, comprising viruses sampled between 1944 and 1977 [7] and classified into the P-I and P-II clades [4], originated after ca. 1654.

To determine a more accurate timescale of VARV evolution, we estimated genome-scale evolutionary rates. An initial regression of root-to-tip genetic distances against year of sampling provided clear evidence for clock-like molecular evolution in VARV (R^2^ = 0.79). Importantly, strong temporal structure (R^2^ = 0.80) was also observed when VD21 was excluded from the regression analysis, indicating that it was not simply the function of a single ancient sequence and that it characterizes VARV evolution as a whole [4, 6, 20]. Similarly clock-like evolution was observed using a Bayesian approach, with extensive overlap in estimates of substitution rates and divergence times under a range of molecular-clock and demographic models (Figure 3; Table S3). Under the model with the narrowest posterior distribution (strict molecular clock and constant population size), the evolutionary rate of VARV is estimated to be between 7.3 and 9.6 × 10^−6^ nucleotide substitutions per site per year (mean of 8.5 × 10^−6^ subs/site/year). This is similar to previous estimates of the evolutionary dynamics of VARV inferred using tip-date based methods on more modern strains only [4, 6, 20] and to rates previously estimated in myxomavirus (another poxvirus) in European rabbits, for which longitudinal sequence data is available for a sampling period of ∼50 years [21]. Finally, we observed overlapping substitution rates (range of credible intervals of 5.6 to 9.5 × 10^−6^ subs/site/year) when VD21 was excluded from the analysis, further suggesting that our estimates of the nucleotide substitution rate are robust.

Assuming a strict molecular clock and a constant population size, we estimate that the VARV strains sampled here (i.e., including VD21) share a common ancestor between 1588 and 1645 (or between 1530 and 1654 under a relaxed molecular clock; see the Supplemental Experimental Procedures for further details). This date corresponds to a time of global exploration and colonization that was most likely central to viral dissemination [22, 23], but before the development of widespread vaccination that began after Edward Jenner utilized the related cowpox virus in 1796 (although, importantly, a related process of “variolation,” or inoculation, had been described in the Islamic world for many centuries, may have been practiced even earlier in China, and was becoming increasingly widespread in both England and other localities during the 18^th^ century [1, 24]). Similarly, according to our molecular timeline and previous molecular-clock analyses [4], the divergence of the P-I and P-II clades occurred between 1734 and 1793, and hence just prior to the development of smallpox vaccination (Figure 3). Interestingly, there is some historical evidence that increasingly widespread inoculation in England during this period may have converted smallpox from a disease largely of adults to one of infants [25]. That the P-II viruses are largely associated with West Africa and the Americas is also compatible with the idea that the divergence of this clade from P-I reflects the movement of people in the context of the 18^th^ century slave trade [4].

It is also striking that the genetic diversity within the P-I and P-II clades has a very recent origin, dating to the end of 19^th^ and the start of 20^th^ century, and hence corresponding to the rise of global smallpox vaccination. Such a phylogenetic pattern strongly suggests that both the P-I and P-II clades experienced a major population bottleneck at this time, most likely leading to the extinction of several unsampled older lineages. Perhaps of greatest note is that the lineage leading to the lower-virulence “alastrim minor” strains is only dated to the 19^th^ century (credible dates of 1855–1885), although few samples are available for analysis. Whether and how increasing levels of vaccination impacted the selection pressures acting on virulence evolution remains uncertain but could be addressed with the acquisition of additional ancient strains.

Finally, to confirm our molecular-clock dating analyses, we estimated substitution rates and divergence times using (1) the lower-density calibrated ^14^C date of 1785–1793 (mean of 1789) of VD21 and (2) a dataset excluding VD21 from all analyses (and hence similar to some datasets used in previous studies [4, 6]). Importantly, these produced very similar estimates to those described above, strongly suggesting that our results are robust (Table S3; Figure 3). For example, the estimated age of the tree excluding VD21, corresponding to the time of separation of P-I and P-II, ranges from 1656–1806 (maximum range of credible intervals across all models; Table S3) and exhibits extensive overlap with the date estimates for the split when VD21 is present. Similarly, although assigning a date of 1789 to VD21 results in a shallower common ancestry for VARV as a whole (mean values of 1666–1686), the remaining divergence times are in accordance with those obtained when VD21 is dated to 1654.

Our characterization of a 17^th^ century mummy strain provides a key calibration point in the epidemiological history of smallpox. Clearly, the evolutionary timescale that we infer, with an origin of the sampled VARV diversity dating to the mid-16^th^ century, is far more recent than some reports of smallpox symptomology [26, 27]. Although molecular-clock approaches can only estimate the timescale of the sampled genetic diversity, apparent conflicts between molecular timelines and symptomology have previously led some authors to reject the use of tip-dated methods to estimate the timescale of smallpox evolution [4]. The most distinctive physical manifestation of smallpox—the pustular rash—has supposedly “definitive” reports in 4^th^ century China, with suggestions that it was present in ancient Egypt and India over 3,500 years ago [1, 2, 4], although in reality it is difficult to distinguish smallpox from chickenpox or measles using historical records alone [26]. Such a discordance between inferred molecular-clock dates and retrospective analyses suggests that if they were indeed due to smallpox, these early cases were caused by virus lineages that were no longer circulating at the point of eradication in the 1970s. Conversely, others have suggested that there is little compelling evidence for epidemic and/or virulent (i.e., high-mortality) smallpox in Europe prior to the 16^th^ century [22], close to our inferred date for the ancestry of VARV. For example, the bills of mortality, the best-known mortality records for Europe at that time, provide the first clear evidence for severe smallpox in London in 1632 [1, 22], shortly before several major European and western Asian epidemics took hold and most likely infected the Lithuanian child studied here [2, 28].

### General Conclusions

To fully resolve the timing of smallpox origins, it will be necessary to determine whether the long branch connecting VARV to the other orthopoxviruses predominantly represents evolution in humans or in other mammals. Although it is tempting to use the evolutionary rates obtained here to date the divergence of VARV from its animal reservoir, the branch lengths leading to the camelpox and taterapox strains are markedly longer than those associated with VARV, such that cross-species transmission may be associated with a change in evolutionary rates that would confound molecular-clock dating, and the host jump to humans could have occurred at any point along this branch. In addition, given the increasing number of animal viruses discovered using metagenomic techniques [29], it is possible that those species currently known to harbor orthopoxvirus are not the true reservoir species for VARV. These uncertainties notwithstanding, our data clearly show that the VARV lineages eradicated during the 20^th^ century had only been in existence for ∼200 years, at a time of rapidly expanding human movement and population size in the face of increasingly widespread inoculation and vaccination.

## Experimental Procedures

All laboratory work was performed in dedicated ancient DNA facilities that form part of the McMaster Ancient DNA Centre. We extracted DNA from a 112.5 mg subsample of soft tissue from VD21, and the resulting extraction was converted into an Illumina sequencing library following a modified protocol and enriched for VARV using a modified MYbaits in-solution capture with a custom VARV bait set (see the Supplemental Experimental Procedures for full details). The enriched library was then mapped to the reference sequence for variola (major) virus (India 1967; GenBank: NC_001611.1), generating a VARV-like genome with an average coverage of 18×. We generated a consensus genome sequence from the reads mapped to sequence NC_001611.1, strictly retaining bases where there was a minimum of 5× coverage and only identifying variants with a frequency of at least 0.9. We also attempted a de novo assembly of the VD21 VARV genome. The final de novo genome was 187,565 bp in length, and the de novo consensus sequence has 97.5% identity across the 185,578 bp to the NC_001611.1 reference VARV sequence.

Full methodologies for both the laboratory and analytical procedures are provided in the Supplemental Experimental Procedures. Ethical approval for this work was granted by the Hospital District of Helsinki under statement no. 164/13/03/00/14. Permission to perform and publish the work reported herein was granted by the World Health Organization's Advisory Committee on Variola Virus Research (ACVVR).

## Author Contributions

D.P.-M., M.F.P., R.J., K.H., and A.S. provided the sample material. M.F.P., S.M., and M.V.E. performed laboratory experiments. A.D. and J.-M.R. developed enrichment bait set for capture. A.T.D., H.P., E.C.H., G.L.S., G.B.G., J.W.S., J.P.B., and S.D. performed the data analysis. D.P.-M., M.H., H.P., O.D., and D.P. provided historical context and smallpox history. J.S. performed radiocarbon dating on the sample. All authors contributed to interpretation, manuscript writing, and editing.

## Accession Numbers

Sequence data have been uploaded to NCBI Sequence Read Archive under BioProject ID NCBI SRA: PRJNA348754.

## Supplemental Information

Document S1. Supplemental Experimental Procedures, Figures S1–S3, and Tables S1–S3

Document S2. Article plus Supplemental Information
