# Supplementary material for: 17th Century Variola Virus Reveals the Recent History of Smallpox
Source: Curr Biol. 2016 Dec 19;26(24):3407–12. doi: 10.1016/j.cub.2016.10.061 (PMC5196022; doi:10.1016/j.cub.2016.10.061)
Supplement: Document S2. Article plus Supplemental Information [file mmc2.pdf]

Current Biology

Report

17th Century Variola Virus Reveals the Recent History of Smallpox

Highlights

- Variola virus genome was reconstructed from a 17th century mummified child
- The archival strain is basal to all 20th century strains, with same gene degradation
- Molecular-clock analyses show that much of variola virus evolution occurred recently

Authors

Ana T. Duggan, Maria F. Perdomo, Dario Piombino-Mascali, ..., Geoffrey L. Smith, Edward C. Holmes, Hendrik N. Poinar

Correspondence

duggana@mcmaster.ca (A.T.D.), edward.holmes@sydney.edu.au (E.C.H.), poinarh@mcmaster.ca (H.N.P.)

In Brief

Using ancient DNA sequences of variola virus recovered from the mummified remains of a 17th century child, Duggan et al. reconstruct the evolutionary history of smallpox. With the ancient strain, the genetic diversification of the smallpox virus is found to be more recent than previously supposed and concurrent with the onset of widespread vaccination.

Duggan et al., 2016, Current Biology 26, 3407–3412
December 19, 2016 © 2016 The Authors. Published by Elsevier Ltd.
http://dx.doi.org/10.1016/j.cub.2016.10.061

CellPress

Current Biology
Report

CellPress

17th Century Variola Virus Reveals the Recent History of Smallpox

Ana T. Duggan,[1,19,*] Maria F. Perdomo,[2,19] Dario Piombino-Mascali,[3,19] Stephanie Marciniak,[1] Debi Poinar,[1] Matthew V. Emery,[1] Jan P. Buchmann,[4] Sebastian Duchêne,[4,5] Rimantas Jankauskas,[3] Margaret Humphreys,[6] G. Brian Golding,[7] John Southon,[8] Alison Devault,[9] Jean-Marie Rouillard,[9,10] Jason W. Sahl,[11] Olivier Dutour,[12,13] Klaus Hedman,[2,14] Antti Sajantila,[15] Geoffrey L. Smith,[16] Edward C. Holmes,[4,*] and Hendrik N. Poinar[1,7,17,18,20,*]
[1]McMaster Ancient DNA Centre, Department of Anthropology, McMaster University, Hamilton, ON L8S 4L8, Canada
[2]Department of Virology, University of Helsinki, Helsinki 00014, Finland
[3]Department of Anatomy, Histology, and Anthropology, Faculty of Medicine, Vilnius University, Vilnius 03101, Lithuania
[4]Marie Bashir Institute for Infectious Diseases and Biosecurity, Charles Perkins Centre, School of Life and Environmental Sciences and Sydney Medical School, The University of Sydney, Sydney, NSW 2145, Australia
[5]Department of Biochemistry and Molecular Biology, Bio21 Molecular Science and Biotechnology Institute, The University of Melbourne, Melbourne, VIC 3010, Australia
[6]Department of History, Duke University, Durham, NC 27708-0719, USA
[7]Department of Biology, McMaster University, Hamilton, ON L8S 4L8, Canada
[8]Keck Carbon Cycle Accelerator Mass Spectrometer, Earth Systems Science Department, University of California, Irvine, CA 92697-3100, USA
[9]MYcroarray, Ann Arbor, MI 48105, USA
[10]Department of Chemical Engineering, University of Michigan, Ann Arbor, MI 48109-2136, USA
[11]Center for Microbial Genetics and Genomics, Northern Arizona University, Flagstaff, AZ 86011-4073, USA
[12]Laboratoire d'Anthropologie Biologique Paul Broca, Ecole Pratique des Hautes Etudes, PSL Research University, Paris 75014, France
[13]PACEA, CNRS, Université de Bordeaux, Pessac 33615, France
[14]Helsinki University Hospital, Helsinki 00029, Finland
[15]Department of Forensic Medicine, University of Helsinki, Helsinki 00014, Finland
[16]Department of Pathology, University of Cambridge, Cambridge CB2 1QP, UK
[17]Michael G. DeGroote Institute for Infectious Disease Research, McMaster University, Hamilton, ON L8S 4L8, Canada
[18]Humans and the Microbiome Program, Canadian Institute for Advanced Research, Toronto, ON M5G 1Z8, Canada
[19]Co-first author
[20]Lead Contact
*Correspondence: duggana@mcmaster.ca (A.T.D.), edward.holmes@sydney.edu.au (E.C.H.), poinarh@mcmaster.ca (H.N.P.)
http://dx.doi.org/10.1016/j.cub.2016.10.061

SUMMARY

Smallpox holds a unique position in the history of medicine. It was the first disease for which a vaccine was developed and remains the only human disease eradicated by vaccination. Although there have been claims of smallpox in Egypt, India, and China dating back millennia [1–4], the timescale of emergence of the causative agent, variola virus (VARV), and how it evolved in the context of increasingly widespread immunization, have proven controversial [4–9]. In particular, some molecular-clock-based studies have suggested that key events in VARV evolution only occurred during the last two centuries [4–6] and hence in apparent conflict with anecdotal historical reports, although it is difficult to distinguish smallpox from other pustular rashes by description alone. To address these issues, we captured, sequenced, and reconstructed a draft genome of an ancient strain of VARV, sampled from a Lithuanian child mummy dating between 1643 and 1665 and close to the time of several documented European epidemics [1, 2, 10]. When compared to vaccinia virus, this archival strain contained the same pattern of gene degradation as 20th century VARVs, indicating that such loss of gene function had occurred before ca. 1650. Strikingly, the mummy sequence fell basal to all currently sequenced strains of VARV on phylogenetic trees. Molecular-clock analyses revealed a strong clock-like structure and that the timescale of smallpox evolution is more recent than often supposed, with the diversification of major viral lineages only occurring within the 18th and 19th centuries, concomitant with the development of modern vaccination.

RESULTS AND DISCUSSION

In an attempt to reveal the evolutionary history of smallpox (variola virus, VARV), we sampled the partial mummified remains of a young child of undetermined sex found within the crypt of the Dominican Church of the Holy Spirit of Vilnius, Lithuania, with no associated artifacts or coffin [11, 12] (Figures 1 and S1). As the bones were covered by soft tissue, it was difficult to obtain a precise age at death, although it is thought to be between 2 and 4 years [13]. Radiocarbon dating of the sample yielded a ^{14}C age

This is an open access article under the CC BY license (http://creativecommons.org/licenses/by/4.0/).

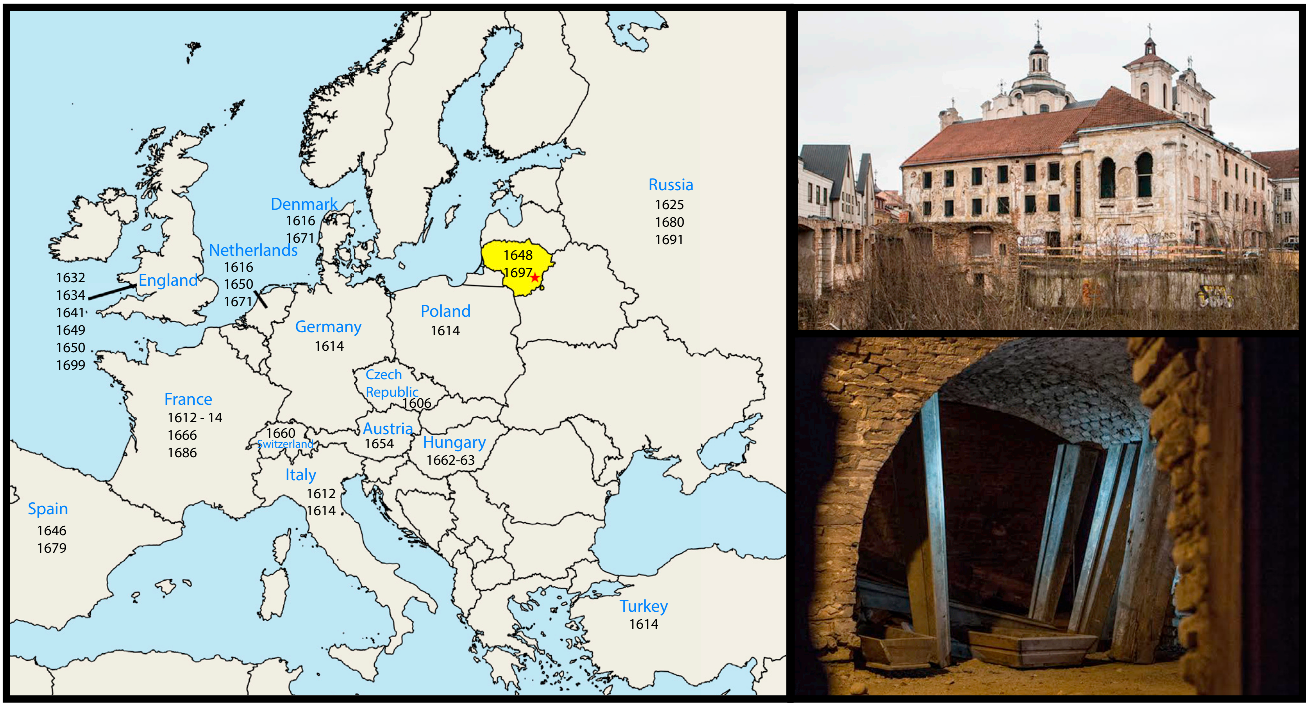

Figure 1. Dominican Church of the Holy Spirit, Vilnius

Left: Lithuania is shaded in yellow, with the red star indicating the city of Vilnius, the location of the Dominican Church of the Holy Spirit where the VD21 specimen was found and dated to approximately 1654. Dates in black indicate known smallpox outbreaks in nearby countries during the 17th century [1, 2]. Top right: the Dominican Church of the Holy Spirit in Vilnius, Lithuania. Bottom right: the crypt where the child mummy was located. See also Figure S1 and Table S1.

of 250 ± 15 BP, which at 2 SDs calibrates to 1643–1665 AD (relative probability, p = 0.93) or 1785–1793 (p = 0.07) (Table S1; Figure S2). The older calibrated age range, which contains the bulk of the probability density function, agrees with historical sources that place this during the Russian occupation of 1655–1661 [12], as well as the reported presence of endemic smallpox within Lithuania. Our older date is also supported by a Bayesian molecular-clock analysis, which gave mean sampling time estimates of 1691 and 1665 under constant size and Bayesian skygrid demographic models, respectively (see the Supplemental Experimental Procedures for further details).

BLAST analysis of a library, enriched for an unrelated target (JC polyomavirus), indicated that of 0.03% of the 1.3 million hits assigned to viruses, 47% (198 reads) were top hits to VARV. To confirm the presence of this virus, we enriched the library using a custom in-solution bait set targeting publicly available strains of VARV and sequenced 845,594 reads (Supplemental Experimental Procedures). We were able to map 43,243 reads to a reference strain of variola (major) virus (India 1967; GenBank: NC_001611.1) and from this reconstructed an ancient VARV genome at an average of 18× coverage (range 0×–60×) (Figure 2). Additionally, through de novo assembly, we reconstructed a draft viral genome that is 187,565 bp in length and contains all annotated genes found in the VARV reference sequence (Supplemental Experimental Procedures). We investigated the synteny in our draft genome by aligning it to the variola major virus reference genome sequence [15]. This revealed that the genome of VD21 shows no major rearrangements and a strong conservation in gene content and arrangement with all other VARVs isolated in the 20th century (Figure 2).

Overall, we identified and confirmed a total of 716 nucleotide substitutions relative to the VARV reference sequence by eye and using NASP [16] in conjunction with GATK [17], with a minimum of 5× coverage and 0.9 frequency. We also enriched for, sequenced, and produced a mitochondrial genome at 193× coverage. The mitochondrial haplogroup of VD21 was H2a5b (Supplemental Experimental Procedures). Haplogroup H2 and its descendent lineages are common throughout Europe. The presence of a common European haplogroup, as well as the DNA damage profiles and overall fragment length distribution of the reads mapped to both VARV and the mitochondrial genome, supports the presence of authentic ancient DNA.

VARV genomes are characterized by the fragmentation of several genes, such that they are non-functional, even though their homologs are intact in vaccinia virus (VACV; GenBank: NC_006998.1) and in other orthopoxviruses [18]. Notably, VD21 exhibited the same pattern of gene disruption in comparison to VACV as more modern strains of VARV, indicating that the loss of gene function during VARV evolution had occurred prior to ca. 1650. However, VD21 also carried a number of additional amino acid substitutions and nonsense or frameshift mutations that might alter gene function, although this remains to be determined.

Phylogenetic analysis of VD21 with 42 additional complete genomes of VARV (Table S2) and the two most closely related orthopoxviruses (camelpox and taterapox) utilized as outgroups

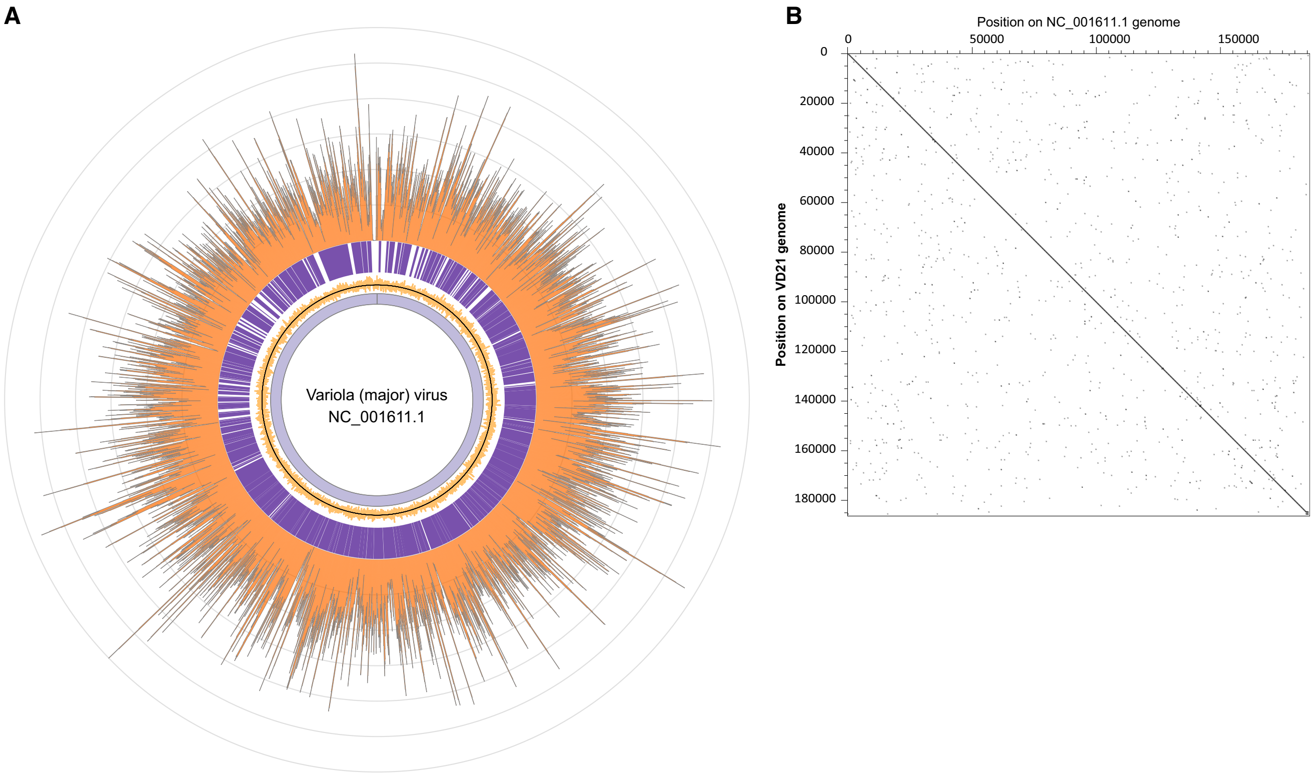

Figure 2. VARV Genome Reconstructed from VD21

(A) Coverage of reference NC_001611.1 variola (major) virus genome. The inner-most circle (light purple) indicates the full 185,578 bp length of the reference, and the inner yellow circle depicts GC content across the reference genome with the genomic average of 32.7% indicated by the thin dark ring. The dark-purple ring indicates the location of annotated genes in the reference. The outer-most ring (dark orange) represents the coverage depth of reads from sample VD21 mapped to the NC_001611.1 reference sequence averaged across 25 bp windows. Average coverage was 18× (minimum 0× to maximum 60×). The concentric gray lines represent intervals of 10× coverage. The plot was constructed with Circos [14].

(B) Conservation of genomic sequence between VD21 and the VARV reference genome NC_001611.1. The plot was constructed with Dotter [15].

See also Figure S2.

clearly placed our ancient strain basal to all previously sequenced VARV strains (Figure 3). The divergent phylogenetic position of VD21 is seemingly similar to that observed in a partial VARV sequence obtained from a 300-year-old Siberian mummy [19], although the 718 bp sequence obtained from that sample provided little phylogenetic resolution (Figure S3). The basal phylogenetic position of VD21 clearly indicates that the previously described genetic diversity of VARV, comprising viruses sampled between 1944 and 1977 [7] and classified into the P-I and P-II clades [4], originated after ca. 1654.

To determine a more accurate timescale of VARV evolution, we estimated genome-scale evolutionary rates. An initial regression of root-to-tip genetic distances against year of sampling provided clear evidence for clock-like molecular evolution in VARV (R^2 = 0.79). Importantly, strong temporal structure (R^2 = 0.80) was also observed when VD21 was excluded from the regression analysis, indicating that it was not simply the function of a single ancient sequence and that it characterizes VARV evolution as a whole [4, 6, 20]. Similarly clock-like evolution was observed using a Bayesian approach, with extensive overlap in estimates of substitution rates and divergence times under a range of molecular-clock and demographic models (Figure 3; Table S3). Under the model with the narrowest posterior distribution (strict molecular clock and constant population size), the evolutionary rate of VARV is estimated to be between 7.3 and 9.6×10^{-6} nucleotide substitutions per site per year (mean of 8.5×10^{-6} subs/site/year). This is similar to previous estimates of the evolutionary dynamics of VARV inferred using tip-date based methods on more modern strains only [4, 6, 20] and to rates previously estimated in myxomavirus (another poxvirus) in European rabbits, for which longitudinal sequence data is available for a sampling period of ~50 years [21]. Finally, we observed overlapping substitution rates (range of credible intervals of 5.6 to 9.5×10^{-6} subs/site/year) when VD21 was excluded from the analysis, further suggesting that our estimates of the nucleotide substitution rate are robust.

Assuming a strict molecular clock and a constant population size, we estimate that the VARV strains sampled here (i.e., including VD21) share a common ancestor between 1588 and 1645 (or between 1530 and 1654 under a relaxed molecular clock; see the Supplemental Experimental Procedures for further details). This date corresponds to a time of global exploration and colonization that was most likely central to viral dissemination [22, 23], but before the development of widespread vaccination that began after Edward Jenner utilized the related cowpox virus in 1796 (although, importantly, a related process of

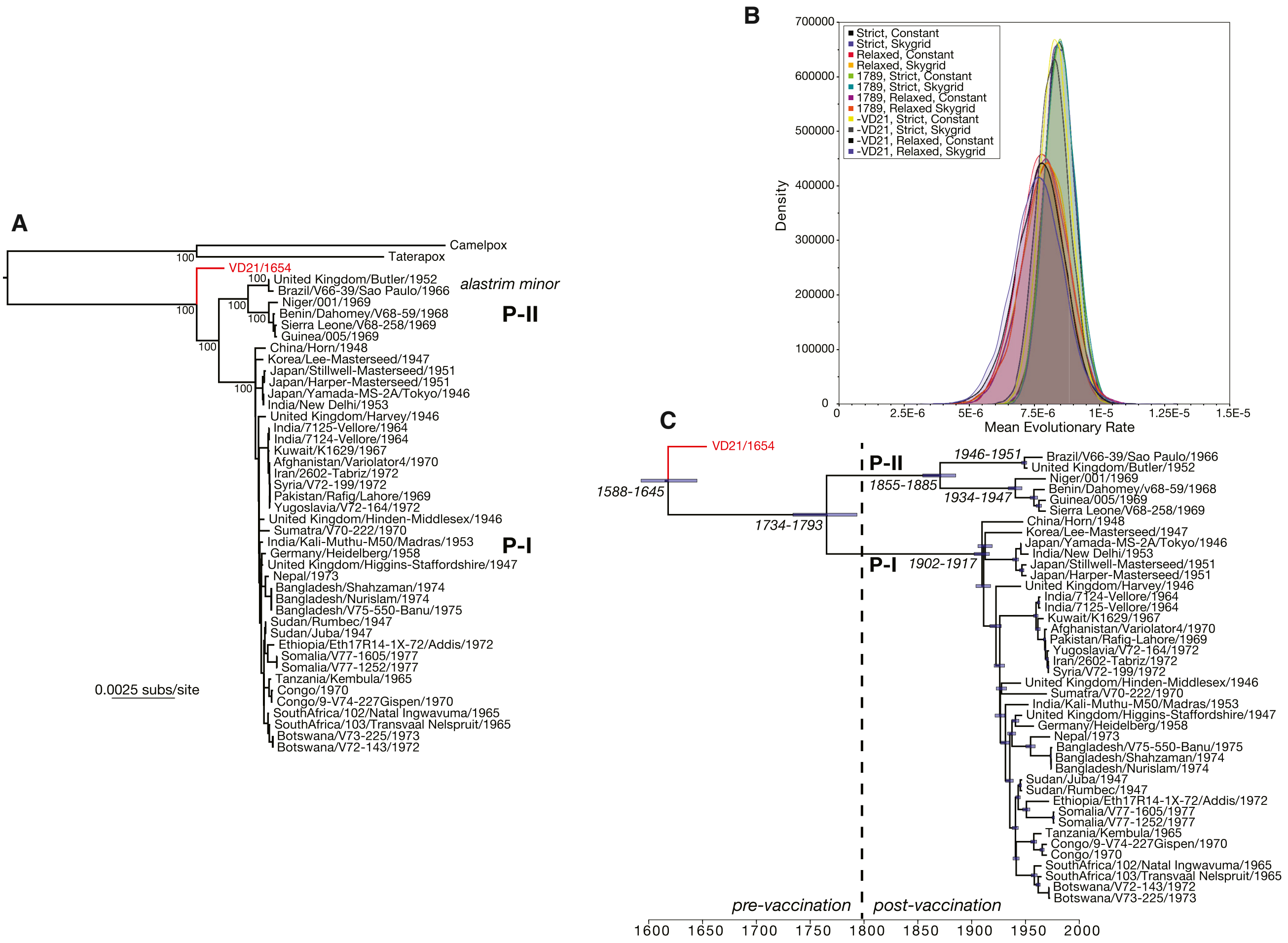

Figure 3. Evolutionary History of VARV

(A) Maximum-likelihood phylogeny of 43 strains of VARV rooted using homologous sequences from camels (camelpox) and African gerbils (taterapox), which are the orthopoxviruses most closely related to VARV. All horizontal branch lengths are drawn to a scale of nucleotide substitutions per site, and bootstrap values are shown for key nodes, with year of sampling shown for the VARV strains. The VD21 strain is shown in red, and the major clades of VARV (P-I and P-II, with the later the containing alastrim minor strains) are marked.

(B) Posterior probability densities of mean evolutionary rate estimates for VARV under different molecular-clock and coalescent models, possible ^{14}C dates for VD21 (i.e., "1789" assumes VD21 is from 1789 rather than 1654), and with VD21 excluded (i.e., "−VD21") from the analysis (see Table S3 for full results).

(C) Maximum clade credibility tree showing the timescale of VARV evolution inferred under a strict molecular clock and a constant population size, with the VD21 lineage shown in red and the 95% credible intervals for a number of other key divergence events also shown. The date (1796) of the introduction of the cowpox-based smallpox vaccine by Jenner, which we include as an historical reference point only, is marked by a hatched line, and it is important to note that a process of smallpox variolation (inoculation) had most likely been in existence for many centuries prior to this.

See also Figure S3 and Tables S2 and S3.

"variolation," or inoculation, had been described in the Islamic world for many centuries, may have been practiced even earlier in China, and was becoming increasingly widespread in both England and other localities during the 18th century [1, 24]). Similarly, according to our molecular timeline and previous molecular-clock analyses [4], the divergence of the P-I and P-II clades occurred between 1734 and 1793, and hence just prior to the development of smallpox vaccination (Figure 3). Interestingly, there is some historical evidence that increasingly widespread inoculation in England during this period may have converted smallpox from a disease largely of adults to one of infants [25]. That the P-II viruses are largely associated with West Africa and the Americas is also compatible with the idea that the divergence of this clade from P-I reflects the movement of people in the context of the 18th century slave trade [4].

It is also striking that the genetic diversity within the P-I and P-II clades has a very recent origin, dating to the end of 19th and the start of 20th century, and hence corresponding to the rise of global smallpox vaccination. Such a phylogenetic pattern strongly suggests that both the P-I and P-II clades experienced a major population bottleneck at this time, most likely leading to the extinction of several unsampled older lineages. Perhaps of greatest note is that the lineage leading to the lower-virulence "alastrim minor" strains is only dated to the 19th century (credible dates of 1855–1885), although few samples are available for analysis. Whether and how increasing levels of vaccination

impacted the selection pressures acting on virulence evolution remains uncertain but could be addressed with the acquisition of additional ancient strains.

Finally, to confirm our molecular-clock dating analyses, we estimated substitution rates and divergence times using (1) the lower-density calibrated ^{14}C date of 1785–1793 (mean of 1789) of VD21 and (2) a dataset excluding VD21 from all analyses (and hence similar to some datasets used in previous studies [4, 6]). Importantly, these produced very similar estimates to those described above, strongly suggesting that our results are robust (Table S3; Figure 3). For example, the estimated age of the tree excluding VD21, corresponding to the time of separation of P-I and P-II, ranges from 1656–1806 (maximum range of credible intervals across all models; Table S3) and exhibits extensive overlap with the date estimates for the split when VD21 is present. Similarly, although assigning a date of 1789 to VD21 results in a shallower common ancestry for VARV as a whole (mean values of 1666–1686), the remaining divergence times are in accordance with those obtained when VD21 is dated to 1654.

Our characterization of a 17th century mummy strain provides a key calibration point in the epidemiological history of smallpox. Clearly, the evolutionary timescale that we infer, with an origin of the sampled VARV diversity dating to the mid-16th century, is far more recent than some reports of smallpox symptomology [26, 27]. Although molecular-clock approaches can only estimate the timescale of the sampled genetic diversity, apparent conflicts between molecular timelines and symptomology have previously led some authors to reject the use of tip-dated methods to estimate the timescale of smallpox evolution [4]. The most distinctive physical manifestation of smallpox—the pustular rash—has supposedly "definitive" reports in 4th century China, with suggestions that it was present in ancient Egypt and India over 3,500 years ago [1, 2, 4], although in reality it is difficult to distinguish smallpox from chickenpox or measles using historical records alone [26]. Such a discordance between inferred molecular-clock dates and retrospective analyses suggests that if they were indeed due to smallpox, these early cases were caused by virus lineages that were no longer circulating at the point of eradication in the 1970s. Conversely, others have suggested that there is little compelling evidence for epidemic and/or virulent (i.e., high-mortality) smallpox in Europe prior to the 16th century [22], close to our inferred date for the ancestry of VARV. For example, the bills of mortality, the best-known mortality records for Europe at that time, provide the first clear evidence for severe smallpox in London in 1632 [1, 22], shortly before several major European and western Asian epidemics took hold and most likely infected the Lithuanian child studied here [2, 28].

General Conclusions

To fully resolve the timing of smallpox origins, it will be necessary to determine whether the long branch connecting VARV to the other orthopoxviruses predominantly represents evolution in humans or in other mammals. Although it is tempting to use the evolutionary rates obtained here to date the divergence of VARV from its animal reservoir, the branch lengths leading to the camelpox and taterapox strains are markedly longer than those associated with VARV, such that cross-species transmission may be associated with a change in evolutionary rates that would confound molecular-clock dating, and the host jump to humans could have occurred at any point along this branch. In addition, given the increasing number of animal viruses discovered using metagenomic techniques [29], it is possible that those species currently known to harbor orthopoxvirus are not the true reservoir species for VARV. These uncertainties notwithstanding, our data clearly show that the VARV lineages eradicated during the 20th century had only been in existence for ~200 years, at a time of rapidly expanding human movement and population size in the face of increasingly widespread inoculation and vaccination.

EXPERIMENTAL PROCEDURES

All laboratory work was performed in dedicated ancient DNA facilities that form part of the McMaster Ancient DNA Centre. We extracted DNA from a 112.5 mg subsample of soft tissue from VD21, and the resulting extraction was converted into an Illumina sequencing library following a modified protocol and enriched for VARV using a modified MYbaits in-solution capture with a custom VARV bait set (see the Supplemental Experimental Procedures for full details). The enriched library was then mapped to the reference sequence for variola (major) virus (India 1967; GenBank: NC_001611.1), generating a VARV-like genome with an average coverage of 18×. We generated a consensus genome sequence from the reads mapped to sequence NC_001611.1, strictly retaining bases where there was a minimum of 5× coverage and only identifying variants with a frequency of at least 0.9. We also attempted a de novo assembly of the VD21 VARV genome. The final de novo genome was 187,565 bp in length, and the de novo consensus sequence has 97.5% identity across the 185,578 bp to the NC_001611.1 reference VARV sequence.

Full methodologies for both the laboratory and analytical procedures are provided in the Supplemental Experimental Procedures. Ethical approval for this work was granted by the Hospital District of Helsinki under statement no. 164/13/03/00/14. Permission to perform and publish the work reported herein was granted by the World Health Organization's Advisory Committee on Variola Virus Research (ACVVR).

ACCESSION NUMBERS

Sequence data have been uploaded to NCBI Sequence Read Archive under BioProject ID NCBI SRA: PRJNA348754.

SUPPLEMENTAL INFORMATION

Supplemental Information includes Supplemental Experimental Procedures, three figures, and three tables and can be found with this article online at http://dx.doi.org/10.1016/j.cub.2016.10.061.

AUTHOR CONTRIBUTIONS

D.P.-M., M.F.P., R.J., K.H., and A.S. provided the sample material. M.F.P., S.M., and M.V.E. performed laboratory experiments. A.D. and J.-M.R. developed enrichment bait set for capture. A.T.D., H.P., E.C.H., G.L.S., G.B.G., J.W.S., J.P.B., and S.D. performed the data analysis. D.P.-M., M.H., H.P., O.D., and D.P. provided historical context and smallpox history. J.S. performed radiocarbon dating on the sample. All authors contributed to interpretation, manuscript writing, and editing.

ACKNOWLEDGMENTS

M.F.P. and K.H. are funded by the Helsinki University Hospital Research and Education Fund, the Finnish-Norwegian Medical Foundation, the Academy of Finland (grant no. 1257964), the Medical Society of Finland, the Sigrid Jusélius Foundation, and the Jane and Aatos Erkko Foundation. D.P.-M. has been supported by the Education Exchange Support Foundation, Ministry of Education and Science, Republic of Lithuania. G.L.S. is a Wellcome Trust Principal Research Fellow. E.C.H. is funded by an NHMRC Australia Fellowship

(grant no. GNT1037231). A.T.D., E.C.H., and H.P. are supported by NHMRC grant GNT1065106. H.P. is supported by a Canada Research Chair, NSERC, SSHRC, CIFAR, and McMaster University. We thank current and former members of the McMaster Ancient DNA Centre, C. Pepperell, and I.H. for their input. We are especially grateful to Agnius Urbanavičius, Justina Kozakaitė, and Daumantas Liekis for their precious support during this research. A.D. and J.-M.R. are both employed at MYcroarray and provided the bait set used here. We thank the Michael G. DeGroote Institute for Infectious Disease Research (IIDR) for generous seed funding for this work.

Received: September 26, 2016
Revised: October 20, 2016
Accepted: October 31, 2016
Published: December 8, 2016

REFERENCES

1. Fenner, F., Henderson, D., Arita, I., Jezek, Z., and Ladnyi, I. (1988). Smallpox and Its Eradication (World Health Organization).
2. Hopkins, D. (2002). The Greatest Killer: Smallpox in History (University of Chicago Press).
3. McNeill, W. (2010). Plagues and Peoples (Anchor).
4. Li, Y., Carroll, D.S., Gardner, S.N., Walsh, M.C., Vitalis, E.A., and Damon, I.K. (2007). On the origin of smallpox: correlating variola phylogenics with historical smallpox records. Proc. Natl. Acad. Sci. USA *104*, 15787–15792.
5. Babkin, I.V., and Babkina, I.N. (2012). A retrospective study of the orthopoxvirus molecular evolution. Infect. Genet. Evol. *12*, 1597–1604.
6. Babkin, I.V., and Babkina, I.N. (2015). The origin of the variola virus. Viruses *7*, 1100–1112.
7. Esposito, J.J., Sammons, S.A., Frace, A.M., Osborne, J.D., Olsen-Rasmussen, M., Zhang, M., Govil, D., Damon, I.K., Kline, R., Laker, M., et al. (2006). Genome sequence diversity and clues to the evolution of variola (smallpox) virus. Science *313*, 807–812.
8. Hughes, A.L., Irausquin, S., and Friedman, R. (2010). The evolutionary biology of poxviruses. Infect. Genet. Evol. *10*, 50–59.
9. Shchelkunov, S.N. (2009). How long ago did smallpox virus emerge? Arch. Virol. *154*, 1865–1871.
10. Paulet, J.J. (1768). Histoire de la Petite Vérole: Avec les Moyens d'en Préserver les Enfans et d'en Arrêter la Contagion en France, *Volume 1* (Ganeau).
11. Piombino-Mascali, D., and Jankauskas, R. (2014). Mummies of Lithuania. In Mummies around the World: An Encyclopedia of Mummies in History, Religion, and Popular Culture, M. Cardin, ed. (ABC-CLIO), pp. 246–247.
12. Piombino-Mascali, D., Urbanavičius, A., Daubaras, M., Kozakaitė, J., Miliauskienė, Ž., and Jankauskas, R. (2015). The Lithuanian mummy project: a historical introduction. Lietuvos Archeologija *41*, 131–142.
13. Scheuer, L., and Black, S. (2004). The Juvenile Skeleton (Academic Press).
14. Krzywinski, M., Schein, J., Birol, I., Connors, J., Gascoyne, R., Horsman, D., Jones, S.J., and Marra, M.A. (2009). Circos: an information aesthetic for comparative genomics. Genome Res. *19*, 1639–1645.
15. Sonnhammer, E.L., and Durbin, R. (1995). A dot-matrix program with dynamic threshold control suited for genomic DNA and protein sequence analysis. Gene *167*, GC1–GC10.
16. Sahl, J.W., Lemmer, D., Travis, J., Schupp, J., Gillece, J., Aziz, M., Driebe, E., Drees, K., Hicks, N., Williamson, C., et al. (2016). The Northern Arizona SNP Pipeline (NASP): accurate, flexible, and rapid identification of SNPs in WGS datasets. bioRxiv. http://dx.doi.org/10.1101/037267.
17. McKenna, A., Hanna, M., Banks, E., Sivachenko, A., Cibulskis, K., Kernytsky, A., Garimella, K., Altshuler, D., Gabriel, S., Daly, M., and DePristo, M.A. (2010). The Genome Analysis Toolkit: a MapReduce framework for analyzing next-generation DNA sequencing data. Genome Res. *20*, 1297–1303.
18. Aguado, B., Selmes, I.P., and Smith, G.L. (1992). Nucleotide sequence of 21.8 kbp of variola major virus strain Harvey and comparison with vaccinia virus. J. Gen. Virol. *73*, 2887–2902.
19. Biagini, P., Thèves, C., Balaresque, P., Géraut, A., Cannet, C., Keyser, C., Nikolaeva, D., Gérard, P., Duchesne, S., Orlando, L., et al. (2012). Variola virus in a 300-year-old Siberian mummy. N. Engl. J. Med. *367*, 2057–2059.
20. Firth, C., Kitchen, A., Shapiro, B., Suchard, M.A., Holmes, E.C., and Rambaut, A. (2010). Using time-structured data to estimate evolutionary rates of double-stranded DNA viruses. Mol. Biol. Evol. *27*, 2038–2051.
21. Kerr, P.J., Ghedin, E., DePasse, J.V., Fitch, A., Cattadori, I.M., Hudson, P.J., Tscharke, D.C., Read, A.F., and Holmes, E.C. (2012). Evolutionary history and attenuation of myxoma virus on two continents. PLoS Pathog. *8*, e1002950.
22. Carmichael, A.G., and Silverstein, A.M. (1987). Smallpox in Europe before the seventeenth century: virulent killer or benign disease? J. Hist. Med. Allied Sci. *42*, 147–168.
23. Riley, J.C. (2010). Smallpox and American Indians revisited. J. Hist. Med. Allied Sci. *65*, 445–477.
24. Chang, C.F. (1998). Dispersing the fetal toxin of the body-conceptions of smallpox aetiology in pre-modern China. In Contagion: Perspectives from Pre-modern Societies, L. Conrad, and D. Wujastyk, eds. (Ashgate Publishing Limited), pp. 23–38.
25. Davenport, R., Schwarz, L., and Boulton, J. (2011). The decline of adult smallpox in eighteenth-century London. Econ. Hist. Rev. *64*, 1289–1314.
26. Cunha, B.A. (2004). Smallpox and measles: historical aspects and clinical differentiation. Infect. Dis. Clin. North Am. *18*, 79–100.
27. Moore, J.C. (1815). The History of the Small Pox (Longman, Hurst, Rees, Orme, and Brown).
28. Chang, C.F. (2002). Disease and its impact on politics, diplomacy, and the military: the case of smallpox and the Manchus (1613-1795). J. Hist. Med. Allied Sci. *57*, 177–197.
29. Firth, C., and Lipkin, W.I. (2013). The genomics of emerging pathogens. Annu. Rev. Genomics Hum. Genet. *14*, 281–300.

Current Biology, Volume *26*

Supplemental Information

17th Century Variola Virus Reveals the Recent History of Smallpox

Ana T. Duggan, Maria F. Perdomo, Dario Piombino-Mascali, Stephanie Marciniak, Debi Poinar, Matthew V. Emery, Jan P. Buchmann, Sebastian Duchêne, Rimantas Jankauskas, Margaret Humphreys, G. Brian Golding, John Southon, Alison Devault, Jean-Marie Rouillard, Jason W. Sahl, Olivier Dutour, Klaus Hedman, Antti Sajantila, Geoffrey L. Smith, Edward C. Holmes, and Hendrik N. Poinar

Supplemental Information

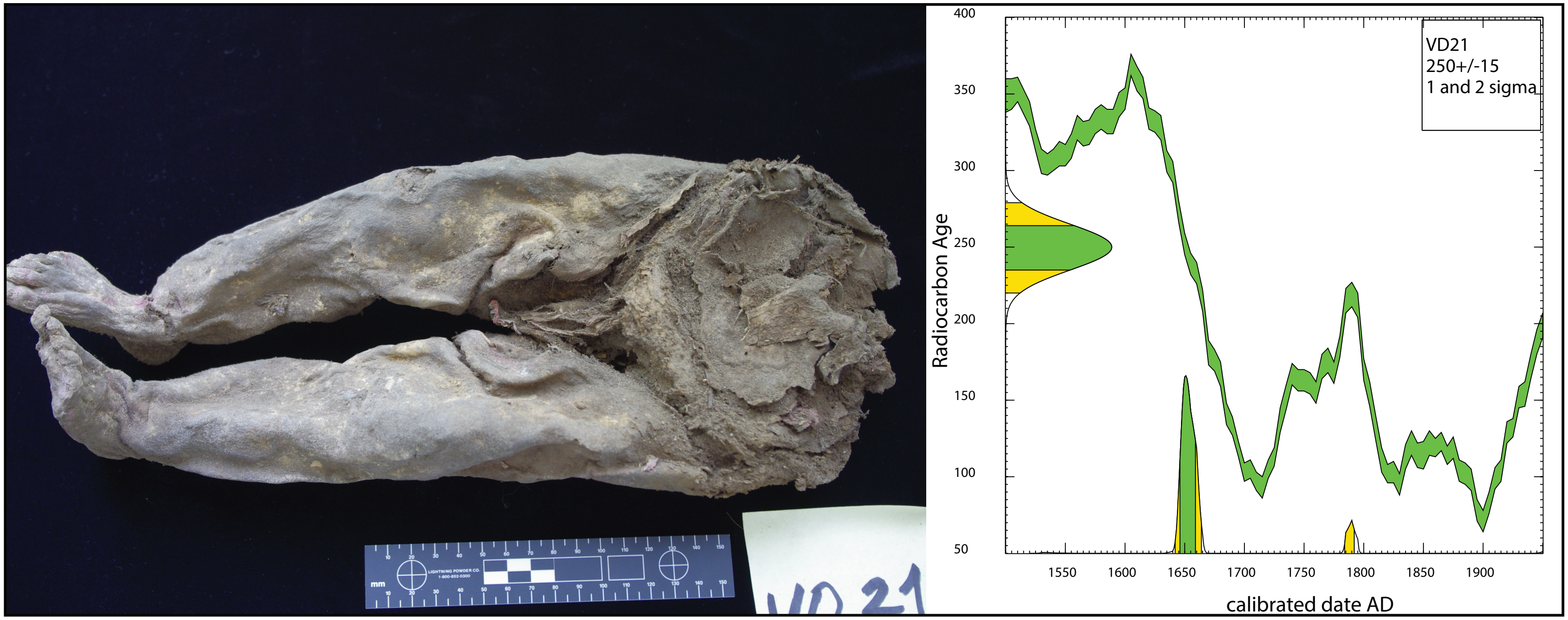

Figure S1. Physical remains and radiocarbon analysis of VD21. Related to Figure 1. Left panel: Mummified remains of a young child of undetermined sex from Lithuania (VD21)**.** Right panel: Radiocarbon calibration for the date on mummy VD21 (Table S1) from Calib7.1, http://calib.qub.ac.uk/calib/ with probability distributions for one and two standard deviations shown in green and yellow, respectively. See text for details.

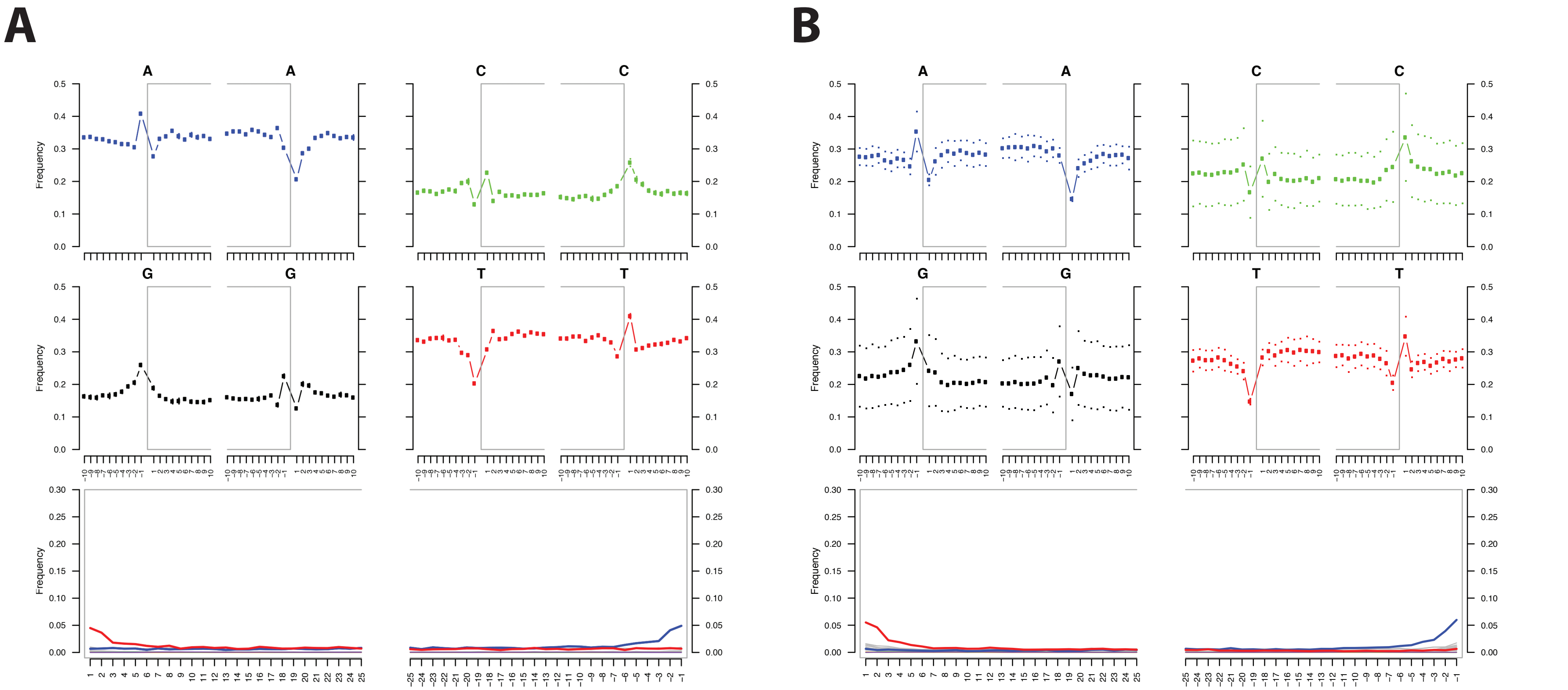

Figure S2. Fragment misincorporation plots. Related to Figure 2. A) Fragment misincorporation of VD21 reads mapped to variola major virus NC_001611.1. B) Fragment misincorporation of VD21 reads mapped to human mitochondrion revised Cambridge Reference Sequence NC_012920.1. In both analyses reads are restricted to those with a minimum length of 35bp and minimum mapping quality of 30. Plots produced by mapDamage 2.0.

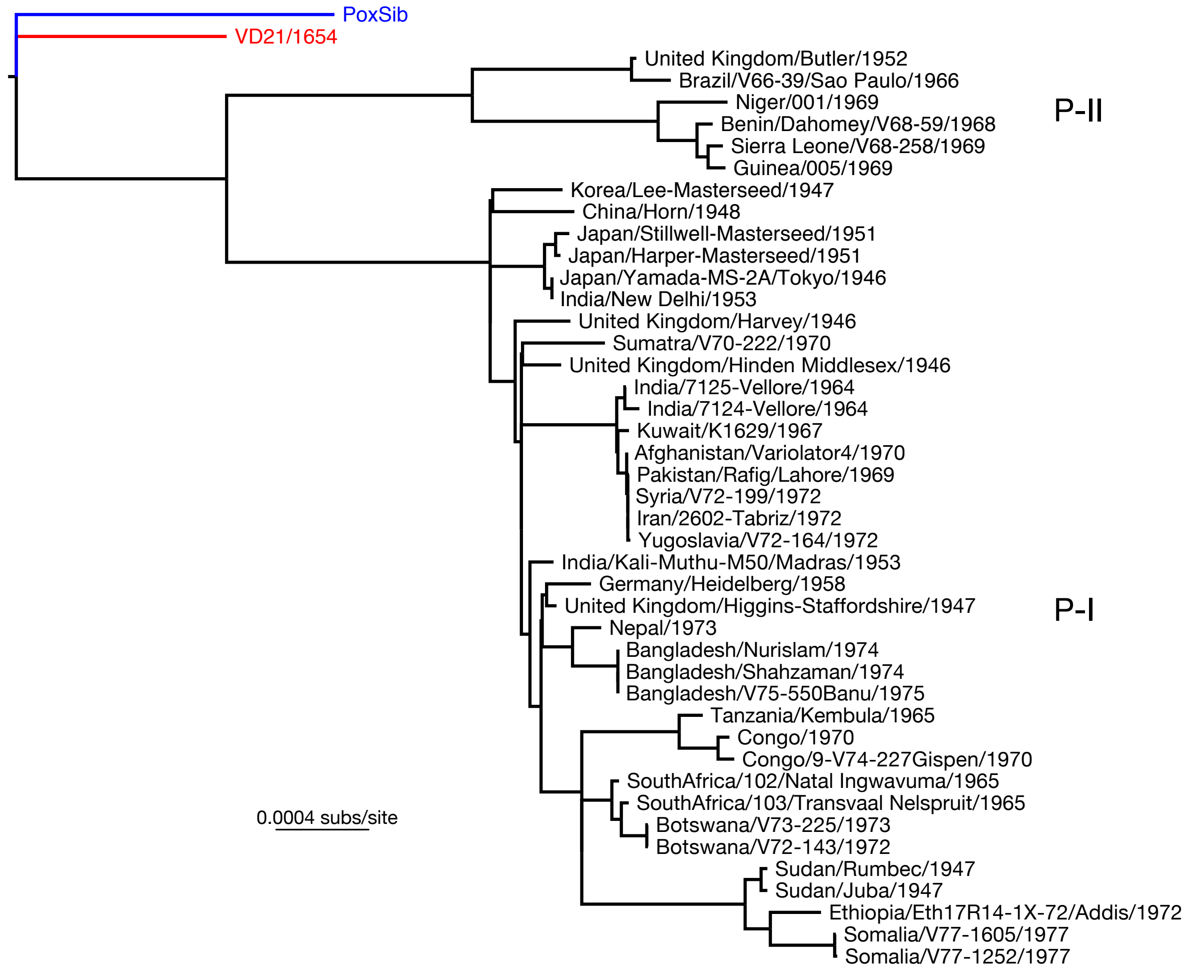

Figure S3. Phylogenetic analysis of VARV including the partial Siberian mummy sequence. Related to Figure 3. The VD21 sequence is shown in red while that from a 300-year-old Siberian mummy ('PoxSib'; 718 bp) is shown in blue. All branch lengths are scaled to the number of nucleotide substitutions per site. The tree is arbitrarily rooted using the VD21 sequence.

Table S1. Radiocarbon measurements of VD21. **Related to Figure 1.** Radiocarbon concentrations are given as fractions of the modern standard, $D^{14}C$, and conventional radiocarbon age, following the conventions of Stuiver and Polach[S1]. Sample preparation backgrounds have been subtracted, based on measurements of ^{14}C-free bone, and the results have been corrected for isotopic fractionation according to the conventions of Stuiver and Polach[S1]. $\delta^{13}C$ and $\delta^{15}N$ values shown were measured to a precision of <0.1‰ and <0.2‰, respectively, on aliquots of ultrafiltered collagen, using a Fisons NA1500NC elemental analyzer/Finnigan Delta Plus isotope ratio mass spectrometer.

**Sample Name**	**VD21**
**UCIAMS**	169464
**$\delta^{13}C$ (‰)**	-19.7 ± 0.1
**Fraction**	0.9692 ± 0.0016
**$D^{14}C$ (‰)**	-30.8 ± 1.6
**^{14}C age**	250 ± 15
**>30kDa collagen yield (%)**	9
**$\delta^{15}N$ (‰)**	15.2
**$\delta^{13}C$ (‰)**	-19.7
**%N**	15.9
**%C**	43.6
**C/N (wt%/wt%)**	2.75
**C/N (atomic)**	3.21

Table S2. Provenance of VARV strains used for bait design and phylogenetic analyses. Related to Figure 3. Strains used for bait design but excluded from phylogenetic analyses are indicated in red.

GenBank accession number	Strain	Year Isolated	Genome Length (bp)
**NC_001611[S2]**	**India/Vector Maharastra E6/1967**	**1967**	**185,578**
**Y16780[S3]**	**Brazil/Garcia alastrim/1966**	**1966**	**186,986**
**X69198[S2]**	**India/Variola virus DNA/1967**	**1967**	**185,578**
DQ441439[S4]	Somalia/V771605/1977	1977	184,170
DQ437592[S4]	Syria/V72-199/1972	1972	185,853
DQ437591[S4]	Sumatra/V70-222/1970	1970	185,449
**DQ437590[S4]**	**Somalia/Variola virus strain/1977**	**1977**	**186,231**
DQ437588[S4]	Nepal/1973	1973	185,654
DQ437587[S4]	Iran/2602_-Tabriz/1972	1972	185,848
DQ437586 [S4]	India/7125-Vellore/1964	1964	186,127
DQ437585[S4]	India/7124-Vellore/1964	1964	186,677
DQ437584[S4]	Germany/Heidelberg/1958	1958	184,900
DQ437583[S4]	Congo/1970	1970	186,553
DQ437582[S4]	China/Horn/1948	1948	186,668
DQ437581[S4]	Bangladesh/V75-550-Banu/1975	1975	185,976
DQ437580[S4]	Afghanistan/Variolator4/1970	1970	185,855
DQ441448[S4]	Yugoslavia/V72-164/1972	1972	185,851
DQ441447[S4]	United_Kingdom/Butler/1952	1952	188,251
DQ441446[S4]	United_Kingdom/Higgins-Staffordshire/1947	1947	185,026
DQ441445[S4]	United_Kingdom/Hinden-Middlesex/1946	1946	186,096
DQ441444[S4]	United_Kingdom/Harvey/1946	1946	185,771
DQ441443[S4]	Tanzania/Kembula/1965	1965	185,826
**DQ441442[S4]**	**Sumatra/V70-228/1970**	**1970**	**185,405**
DQ441441[S4]	Sudan/Rumbec/1947	1947	186,415
DQ441440[S4]	Sudan/Juba/1947	1947	186,284
DQ441438[S4]	Somalia/V77-1252/1977	1977	184,191
DQ441437[S4]	SierraLeone/V68-258/1969	1969	187,014
DQ441436[S4]	SouthAfrica/103/Transvaal Nelspruit/1965	1965	185,881
DQ441435[S4]	SouthAfrica/102/Natal Ingwavuma/1965	1965	186,050
DQ441434[S4]	Niger/001/1969	1969	186,942
DQ441433[S4]	Kuwait/K1629/1967	1967	185,853
DQ441432[S4]	Korea/Lee-Masterseed/1947	1947	186,383
DQ441431[S4]	Japan/Stillwell-Masterseed/1951	1951	186,115
DQ441430[S4]	Japan/Harper-Masterseed/1951	1951	186,180
DQ441429[S4]	Japan/Yamada-MS-2A/Tokyo/1946	1946	186,662
DQ441428[S4]	India/New_Delhi/1953	1953	186,662
DQ441427[S4]	India/Kali-Muthu-M50/Madras/1953	1953	186,108
DQ441426[S4]	Guinea/005/1969	1969	186,883
DQ441425[S4]	Ethiopia/Eth17R14-1X72/Addis/1972	1972	186,648
**DQ441424[S4]**	**Ethiopia/Eth16R14-1X72/Addis/1972**	**1972**	**186,648**
DQ441423[S4]	Congo/9-V74-227Gispen/1970	1970	186,652
DQ441421[S4]	Bangladesh/Shahzaman/1974	1974	186,293
DQ441420[S4]	Banglasdesh/Nurislam/1974	1974	186,293
DQ441419[S4]	Brazil/V66-39/Sao Paulo/1966	1966	188,062
DQ441418[S4]	Botswana/V73-225/1973	1973	185,931
DQ441417[S4]	Botswana/V72-143/1972	1972	185,931
DQ441416[S4]	Benin/Dahomey/V68-59/1968	1968	187,070
DQ437589[S4]	Pakistan/Rafig/Lahore/1969	1969	185,865
**L22579[S5]**	**Bangladesh/Variola major virus/1975**	**1975**	**186,103**

Table S3. Results of the BEAST analysis of evolutionary rates and time-scale in VARV. Related to Figure 3.

Data Set and Model	Substitution Rate ($x10^{-6}$ subs/site/year)	tMRCA	Coefficient of Variation (CoV)	Posterior Probability
[a]Strict, Constant	8.4<br>(7.3 – 9.6)	1617<br>(1588 – 1645)	-	-260114.44<br>(-260127.60 – -260103.24)
Strict, Skygrid	8.5<br>(7.3 – 9.6)	1618<br>(1589 – 1647)	-	-260111.39<br>(-260205.62 – -260010.42)
Relaxed, Constant	7.7<br>(6.1 – 9.4)	1592<br>(1530 – 1654)	0.22<br>(0.06 – 0.38)	-260474.03<br>(-260490.12 – -260458.25)
Relaxed, Skygrid	7.9<br>(6.1 – 9.6)	1598<br>(1533 – 1654)	0.22<br>(0.06 – 0.39)	-260481.05<br>(-260577.88 – -260378.86)
[b]1789, Strict, Constant	8.5<br>(7.3 – 9.7)	1685<br>(1656 – 1715)	-	-260115.14<br>(-260127.62 – -260103.20)
1789, Strict, Skygrid	8.5<br>(7.4 – 9.7)	1686<br>(1659 – 1716)	-	-260113.63<br>(-260213.11 – -260014.21)
1789, Relaxed, Constant	7.9<br>(6.2 – 9.7)	1666<br>(1598 – 1724)	0.22<br>(0.06 – 0.37)	-260474.80<br>(-260490.93 – -260458.69)
1789, Relaxed, Skygrid	7.9<br>(6.0 – 9.6)	1666<br>(1595 – 1723)	0.22<br>(0.07 – 0.39)	-260473.69<br>(-260579.56 – -260373.19)
[c]-VD21, Strict, Constant	8.3<br>(7.1 – 9.4)	1761<br>(1730 – 1792)	-	-257675.95<br>(-257688.88 – -257664.76)
-VD21, Strict, Skygrid	8.2<br>(7.1 – 9.5)	1760<br>(1727 – 1792)	-	-257710.78<br>(-257769.46 – -257622.07)
-VD21, Relaxed, Constant	7.7<br>(5.8 – 9.5)	1739<br>(1656 – 1805)	0.22<br>(0.01 – 0.39)	-258027.25<br>(-258043.76 – -258011.69)
-VD21, Relaxed, Skygrid	7.6<br>(5.6 – 9.5)	1741<br>(1659 – 1807)	0.24<br>(0.03 – 0.43)	-258062.68<br>(-258119.66 – -257963.75)

[a]BEAST analysis based on a mean tip-date of 1654 for VD21.
[b]BEAST analysis based on a mean tip-date of 1789 for VD21.
[c]BEAST analysis with VD21 excluded.
Strict = strict molecular clock; Relaxed = relaxed (uncorrelated lognormal) molecular clock; Constant = constant population size; Skygrid = Bayesian skygrid

Supplemental Experimental Procedures

Information on the Sampling Site

The specimen investigated in this paper, denoted VD21, comes from the Dominican Church of the Holy Spirit of Vilnius, Lithuania. In the crypt of this church, formed by a central, main space and eight side rooms, hundreds of bodies belonging to the clergy, the nobility, and the middle-class had been stored in wooden coffins for burial. Over time, most of these bodies were accumulated in one of the side rooms, where they eventually became skeletonized, with the exception of 23 mummies that are still in a pristine state of preservation[S6]. Historically, the current building appears to be the result of a reconstruction carried out in the aftermath of the Russian occupation of 1655-1661. Furthermore, in 1844 the priory annexed to the church was confiscated by the Russians and transferred to the city's authority[S7]. These dates are in agreement with the calibrated radiocarbon age of 1654±15 CE.

Historical Context – Lithuania, 1650

The main period of use of the Dominican Church can be placed between the creation of the Polish-Lithuanian Commonwealth in 1569, and its final partition carried out in 1795 by the Russians, the Prussians, and the Austrians[S8]. This event determined the annexation of most of the Lithuanian territory to the Russian empire until 1915, with just a short Napoleonic interlude in 1812. While it is recognized that the Polish-Lithuanian period was a culturally florid one, ceding the way for inclusion within central Europe, it should be noted that several factors were responsible for a general instability in Lithuanian society. These included the Swedish and Russian invasions of 1654-1667, and the Great Northern War of 1700-1721[S8]. Cities and towns often had to pay a contribution to their own or foreign armies, also experiencing robberies and pillaging. The country faced a currency crisis, military taxation, and increasing foreign debts[S9]. Between the 17th and the 18th century, a number of famines and plague outbreaks also ravaged the population. By 1709, a severe pandemic of plague, accompanied by other epidemics such as typhoid fever, typhus, smallpox, and dysentery, had also developed in Vilnius as a consequence of crop failures and an extremely cold winter[S10, S11].

Mummy Specimen

The mummy described in this article was inspected in the summer of 2011 within the framework of the "Lithuanian Mummy Project". The subject consisted of the lower part of a young child's body of undetermined sex. As the bones were covered by soft tissue, it was difficult to ascertain a precise age at death, which is somewhere between 2 and 4 years[S12]. Despite the lack of a large portion of the body, the remaining parts were well preserved and appeared to be desiccated (Figure S1). In all likelihood, the principle behind its preservation, as well as the preservation of the remaining 22 mummies, was a natural dehydration process[S13]. No artifacts or coffin were associated with this mummy.

Radiocarbon Dating

Radiocarbon dating was carried out at the University of California Irvine Keck AMS laboratory on ultrafiltered collagen extracted from the skin sample provided. Since there was a possibility that the mummy had been in contact with soil, a room temperature acid-base-acid treatment (0.5N HCl, 0.05N NaOH, 0.5N HCl) was first applied to remove any contaminating sedimentary carbonate and/or soil humics. The sample was then washed twice in Milli-Q water and treated with 0.01N HCl overnight at 60°C to convert collagen to soluble gelatin, and a high molecular weight (>30kDa) gelatin fraction was selected by ultrafiltration and lyophilized[S14]. Collagen aliquots from the skin sample together with suitable collagen process blanks and standards of known age and isotopic composition were combusted *in vacuo* with CuO in sealed quartz tubes at 900°C, graphitized by iron-catalyzed hydrogen reduction, and measured using standard techniques of Accelerator Mass Spectrometry. Radiocarbon results are expressed as conventional radiocarbon ages and were calibrated using Calib 7.1 (http://calib.qub.ac.uk/calib/) with the IntCal13 data set[S15] (Figure S1; Table S1). Though the isotopic analyses support two potential calibrated ^{14}C age ranges, the higher density (and therefore more likely) date of 1643-1665 is more consistent with other independent lines of evidence (i.e. historical) and thus we feel confident in this assessment. However, even with the additional, lower density sample 1730-1792, our estimates of tMRCA remain similar and thus support the main conclusions in the paper (see below; Table S3).

Mummy Sampling

The laboratory work was performed at McMaster University Ancient DNA Centre (McMaster University, Hamilton, ON, Canada). The ancient clean room (sampling, PCR set-up, extraction) and modern laboratory (PCR amplification, enrichment) are physically separate facilities.

The laboratory workspace was cleaned prior to the sub-sampling of specimen VD21 with a bleach solution followed by UV-irradiated double-distilled water. Disposable scalpel blades were used to remove skin sub-samples and placed in a weighing boat. Small sections were transferred with tweezers to 1.5-mL MAXYmum Recovery PCR tubes. The sub-samples were stored at -20˚C for enzymatic extraction.

DNA Extraction
Extractions were performed using a modified tissue extraction protocol[S16] with 112.5-mg of sub-sampled tissue. The tissue was subject to demineralization in 1.0-mL EDTA (pH 8.0, 0.5M) shaken at 1,000 rpm in a Thermomixer at 22˚C for 24 hours, with removal after centrifugation (16,100 x g for 10 minutes) and storage of the supernatant at -20˚C. Following this, the tissue was subject to enzymatic digestion in a buffer [20 mM Tris-HCl pH 8.0, 5 mM calcium chloride, 50mM dithiothreitol (DTT), 2.5 mM N-phenacylthiazolium bromide (PTB), 1% polyvinylpyrrolidone (PVPD), 0.5% sarcosyl, 20 mg/mL Proteinase K, and nuclease-free water] incubating for approximately 3 hours in a Thermomixer at 55˚C shaken at 1,000 rpm. Following centrifugation (16,100 x g for 5 minutes), the supernatant was removed and stored at -20˚C[S16].

The digested material was subject to organic extraction and purification following an ultra-short recovery protocol[S17], with modifications for the 3.0-mL of supernatant. 1.0-mL of the supernatant was added to a binding buffer solution consisting (in final concentrations) of 5M guanidine hydrochloride, 40% (vol/vol) isopropanol, 0.05% Tween-20, and 90mM sodium acetate (pH 5.2) in a Roche nucleic acid column. The solution was spun for 4 minutes at 1,500 x g, rotated 90 degrees, and spun again for 2 minutes at 1,500 x g. This process was repeated for the second and third 1.0-mL of the supernatant. The column was then removed from the reservoir, placed in a clean 2.0-mL MAXYmum recovery tube, followed by a dry spin at 3,300 x g for 1 minute (flow through discarded). Two washes of 750µL PE Buffer (Qiagen) were performed, centrifuging at 3,300 x g (flow through discarded), with two dry spins at 16,100 x g (rotating the tube 180 degrees after the first spin) and placed in a new 1.5mL collection tube. For elution, 25-µL of buffer EBT (15.0-mL of Buffer EB from Qiagen, plus 7.5µL of Tween-20) was added to the silica membrane, incubated for five minutes at room temperature and centrifuged for 30 seconds at maximum speed. This step was repeated twice for a total of 50-µL of DNA extract, which was then stored at -20˚C.

Library Preparation and Indexing
Double-stranded library preparation was performed as outlined for sequencing on the Illumina platform[S18, S19], with modifications detailed below, using 25-µL of extracts (n=2), and extraction blanks (n=2). The libraries were prepared according to the blunt end repair protocol (without removal of cytosine), and MinElute PCR purification (Qiagen) was used instead of SPRI bead clean-up between library preparation steps (blunt-end repair, adapter ligation, and adapter fill-in). Heat deactivation (80˚C for 20 minutes) was performed after the fill-in stage instead of an additional purification step. The final library volume was 40-µL, which was then used directly as templates for double-indexing.

Post-library indexing. A unique P5 and P7 index combination was added to each library in parallel 40-µl reactions following established protocols for double indexing amplification[S19]. The indexing reactions consisted of the following: library inputs of 20-µL, indexing primers at 150-nM, and KAPA SYBR ® FAST qPCR Master Mix (2X). The samples were amplified for 10 cycles or until reaching plateau, with the following conditions: 95˚C denaturation (5 min), 95˚C (30 sec), 60˚C (45 sec), and a final 60˚C (3 min). Indexed products for each library were purified via MinElute PCR Purification Kit (Qiagen), and then eluted in 25-µl EB.

Quantifying indexed libraries. To quantify the total DNA in all indexed libraries (pre- and post-capture), parallel 10-µL real-time PCR reactions used the primer combination IS5_long_amp.P5 and IS6_ long_amp.P7, KAPA SYBR ® FAST qPCR Master Mix (2X) and 1:1,000 dilutions of each library, with a 425bp-525bp PhiX Control (Illumina) standard serially diluted from 1-nM to 0.0625-pM, to infer the concentration of the libraries. The qPCR conditions were the following: 200nM of each primer, 1X KAPA SYBR ® FAST qPCR Master Mix (2X), 1:1,000 library dilutions (4-µL), PhiX serial dilutions (4-µL) alongside two water blanks and two EBT blanks were subjected to a 95˚C activation step (5 min), followed by 35 cycles of 95˚C (30 sec), 60˚C (45 sec), and a melt curve 60-95˚C, ending with an 8˚C hold (30 sec).

Re-amplification. Additional amplification was performed on the one of the indexed VD21 libraries in a 40-µL reaction consisting of: 4-µL of indexed library template DNA in 14.8-µL of double-distilled water, primer combination IS5_long_amp.P5 and IS6_ long_amp.P7 (150 nM each), and KAPA SYBR ® FAST qPCR Master

Mix (2X). The reaction proceeded as: 95˚C activation (5 min), 10 cycles of 95˚C (30 sec), 60˚C (45 sec), ending with a 60˚C hold (3 min). Following this amplification, the sample was purified with the modified MinElute PCR Purification (Qiagen) protocol, eluting in 25-µL EB.

Identification of Variola Virus Sequencing Reads in VD21

We identified 198 reads that matched variola virus (VARV) in a preliminary BLAST analysis of 5.7M reads sequenced from the enrichment of an unrelated JC polyomavirus bait set. Based on these results we generated a targeted enrichment bait set for VARV.

Variola Virus Capture Bait Design

We compiled the sequences of 49 strains of VARV (Table S2), soft-masked these for simple repeats and low-complexity DNA only using RepeatMasker.org, and aligned them using MAFFT v7[S20] and employing default settings. From these aligned sequences we designed 80nt baits with 8x tiling density (10nt flexible spacing). All redundant baits with 5 or fewer mismatches were collapsed to reduce the total unique bait count, and baits containing any soft-masked sequences were removed. This resulted in a final filtered set of 18,473 unique baits, which was used in all downstream targeted enrichment experiments. This bait set was designed and manufactured at MYcroarray (Ann Arbor, MI) as part of a custom MYbaits® targeted enrichment kit.

Variola Virus Enrichment Strategy

In-solution enrichment was performed according to the manufacturer's protocol (MYcroarray, Ann Arbor, MI), with modifications appropriate for degraded and rare ancient DNA, such as 55˚C hybridization temperature, 16-24h hybridization capture, 100-ng bait concentration, 20-µL of washed beads per reaction, and indexed library inputs of 6-µL (brought to 9-µL using double-distilled water). Two rounds of enrichment were completed with these parameters, with the modification that 9-µL of the purified enriched library was used as the input for the second round of enrichment. The re-amplification after each round of enrichment proceeded in 40-µL reactions using the primer combination IS5_long_amp.P5 and IS6_ long_amp.P7 (150-nM each), and KAPA SYBR ® FAST qPCR Master Mix (2X). The parameters were as previously described: a 95˚C activation (5 min), 12 cycles of 95˚C (30 sec), 60˚C (45 sec), ending with a 60˚C hold (3 min). Following this second (and final) reamplification of the capture library, it was purified with the modified MinElute PCR Purification (Qiagen) protocol described previously, and eluted in 15-µL of EB.

Human Mitochondrial Enrichment Strategy

In-solution enrichment was performed as described above using a bait set designed against a global reference of 197 human mitochondrial genomes (Human Global Panel, MYTObaits, MYcroarray, Ann Arbor, MI).

Sequencing

Sequencing was performed on the Illumina HiSeq 1500 platform at the Farncombe Family Digestive Health Research Institute (McMaster University, Hamilton ON, Canada). The VARV enriched samples were pooled in equimolar ratios and sequenced using 2 x 90 bp read chemistry. The mitochondrial enriched library was sequenced separately, at the same facility, using 2 x 100 bp read chemistry.

Bioinformatic Processing of Sequence Data

CASAVA processed reads were trimmed and merged using the leeHom software package[S21], employing ancient DNA parameters (--ancientdna), and then mapped to the reference sequence of variola (major) virus (GenBank accession NC_001611.1) using a modified version of BWA[S22] [https://github.com/udo-stenzel/network-aware-bwa] with a maximum edit distance of 0.01 (-n 0.01), allowing a maximum of two gap openings (-o 2) and with seeding effectively disabled (-l 16500). Mapped reads were further filtered to those that were either merged or unmerged but properly paired [https://github.com/grenaud/libbam] as well as unique based on both 5' and 3' coordinates [https://github.com/udo-stenzel/biohazard]. For all further analyses, we restricted reads to those where the minimum length of inserts was 35bp and the minimum mapping quality was 30. With these criteria, we retained 43,243 unique reads mapped to the VARV reference sequence (NC_001611.1) from an original 845,594 trimmed and merged reads. The average coverage depth of reads mapped to the VARV reference genome is shown in Figure 2. The mitochondrial enriched libraries were processed in the same manner and mapped to the revised Cambridge Reference Sequence (GenBank accession NC_012920.1)[S23]. From an original 2,927,625 trimmed and merged reads of the mitochondria enriched library, we kept 42,376 unique reads of minimum 35bp length and minimum mapping quality 30.

Ancient DNA Damage Assessment

Fragment length distributions. Fragment length distributions (FLD) for the VARV and mtDNA enriched VD21 libraries are predominantly composed of short fragments, as is typical for ancient DNA libraries. The FLD of all sequenced reads from the VARV enriched library has a mean fragment length of 62.5bp (min 1bp, max 169bp), all unique reads mapped to the variola (major) virus reference sequence (GenBank accession NC_001611.1) have a mean length of 75.4bp (min 1bp, max 169bp) and when restricted to fragments of min 35bp and mapping quality 30, the mean length is 77.4bp (min 35bp, max 169bp). The FLD of all sequenced reads from the mtDNA enriched library has a mean fragment length of 70.6bp (min 1bp, max 191bp), all unique reads mapped to rCRS (GenBank accession NC_012920.1) have a mean length of 68.6bp (min 1bp, max 191bp) and when restricted to fragments of min 35bp and mapping quality 30, the mean length is 76.3bp (min 35bp, max 191bp).

DNA damage profiles. Terminal damage and deamination was measured with mapDamage2.0[S24] and observed to be present at low but consistent frequencies across all molecules mapped to the variola (major) virus reference sequence and the human mitochondrial reference genome (Figure S2).

Human mitochondrial genome

From our mitochondrial enrichment, we reconstructed a human mitochondrial genome at average 195X coverage. We used schmutzi[S25] to call the consensus sequence and estimate contamination within the library, inferred from read length and deamination patterns. Contamination for the mitochondrial enriched library was estimated at 2% and the haplogroup was determined to be H2a5b from Haplogrep[S26] using PhyloTree Build 17[S27].

Variola Virus Genome Reconstruction

We attempted to generate a *de novo* VARV genome from the enriched VD21 library. We first processed the CASAVA generated R1 and R2 fastq files using leeHom[S21]. The trimmed and merged fastq files were then used as input for SPAdes v3.6[S28] wherein merged reads were treated as single-end sequenced reads (--s) and trimmed but unmerged reads were treated as paired-end reads (--pe1 and --pe2). The SPAdes assembly was then evaluated with QUAST[S29] which showed that of the 2021 contigs produced by SPAdes, 26 were >= 1000bp, the largest contig was 17,978bp; total length of all contigs was 216,106bp with a GC content of 35.66%, an N50 of 10,140bp and an N75 of 5,762bp. Blast analysis of these contigs returned hits for 584 of the 2,021, of which 29 had a top hit with the orthopoxviruses and the majority consisted of bacterial species of the phylum and class Actinobacteria, frequently found in soil. Using BWA-MEM[S30], 31 of the contigs were mapped back to the VARV reference sequence, in which the shortest mapped contigs was 58 bp, the longest 17,978 bp, and the total length of the mapped contigs was 187,565 bp.

Genome content. The genome of VD21 shows extensive conservation with the gene content and arrangement of other VARVs isolated in the 20th century. VARV genomes are characterized by the fragmentation of several genes, rendered non-functional, yet intact in vaccinia virus (VACV) or other orthopoxviruses[S31]. VACV, is the model organism for virulence on this virus due to safety concerns with work on variola virus. Notably, some of the intact genes in VACV are known to encode proteins that increase VACV virulence[S32, S33], despite being disrupted in many VARV strains. That these genes were also disrupted in VD21 reveals that loss of gene function associated with the evolution of VARV from an ancestral virus clearly occurred prior to ~1650 CE. Unfortunately, the factors contributing to the exceptional virulence of VARV (major) and the reason why VARV (minor) was less virulent remain unknown and cannot be deduced from nucleotide sequence alone.

Synteny. To detect synteny across the VD21 genome we aligned it to the variola (major) virus (GenBank accession NC_001611.1) using dotter[S34]. In both cases the comparison revealed overall high conservation at the genomic scale with no major rearrangements (Figure 2).

Single Nucleotide Polymorphisms. Nucleotide substitutions between VD21 and the VARV NC_001611.1 reference were scored both manually and with the Northern Arizona SNP Pipeline[S35] (NASP) implementation of GATK[S36] at positions where there was a minimum of 5X coverage and the variant frequency was at least 0.9. All 716 final SNPs were manually confirmed and are distributed quite evenly across the genome.

Evolutionary Analyses

Comparative data set. To place the mummy VD21 sequence in historical context we compared it to 42 complete genome sequences of VARV downloaded from GenBank, primarily sourced from Esposito et al. (2006)[S4]. Only VARV strains with known times of sampling were included and likely duplicate strains were excluded (Table S2). In addition, the sequences of the two most closely related orthopoxviruses (camelpox and taterapox) were included as outgroups to root the tree. Including more divergent orthopoxviruses (such as vaccinia virus) reduces phylogenetic resolution within the VARV group. All sequences were aligned using various routines in MAFFT[S20], with all ambiguously aligned regions then removed using Gblocks[S37]. This resulted in a final data set of size 45 sequences, 175,087 bp in length. For the molecular clock dating analysis, in which the outgroup sequences are not required, the camelpox and taterapox sequences were removed and the sequence alignment and Gblocks-pruning procedure was repeated. This resulted in a data set of 43 sequences, 182,738 bp in length. No evidence for recombination was seen for any sequence within the VARV genome sequence alignment using the RDP, Bootscan and GeneCov methods (with default settings) available in the RDP4 package[S38].

Phylogenetic analysis. For the full (45 sequence) data set a phylogenetic tree was inferred using the maximum likelihood procedure available in PhyML[S39]. This analysis utilized the GTR+I+Γ_4 model of nucleotide substitution and a combination of NNI and SPR branch-swapping. The robustness of individual nodes on the tree was assessed by bootstrap resampling (1000 replicates) using the same substitution model as described above and NNI branch-swapping. An additional phylogenetic analysis was undertaken incorporating the 718 bp partial VARV sequence ("PoxSib") obtained from a Siberian mummy[S40] using the same parameters and search procedure. This revealed that PoxSib and VD21 seemingly share common ancestry, although the short length of the PoxSib sequence necessarily meant there was limited phylogenetic resolution (Figure S3).

Nucleotide substitution rates and the time-scale of VARV evolution. To reveal the time-scale of VARV evolution (i.e. time to most recent common ancestor; tMRCA) we first assessed the degree of clock-like structure in the data using a regression of root-to-tip genetic distances against year of sampling using the TempEst program[S41]. For this analysis the phylogeny was rooted as revealed in the ML analysis with the orthopoxvirus outgroups, such that the VD21 strain formed the basal lineage within the VARV group, but possessing a number of lineage-defining mutations so that it did not fall exactly at the node. Although this analysis revealed strong temporal structure (R^2 = 0.79; correlation coefficient = 0.89), it may have been adversely affected by the early sampling date of the VD21 sequence. Therefore, to determine whether VARV possessed clock-like signal in the absence of VD21, we repeated the root-to-tip regression with the VD21 mummy sequence excluded: this also resulted in a strong and clear temporal structure (R^2 = 0.80; correlation coefficient = 0.89).

We next inferred the rates and dates of viral evolution using the Bayesian Markov chain Monte Carlo (MCMC) approach available in the BEAST package[S42]. For this analysis we again utilized the GTR+I+Γ_4 substitution model, strict and relaxed (uncorrelated lognormal) molecular clocks, and both constant population size and Bayesian skygrid coalescent priors. As the VD21 strain was most likely dated to 1643-1665, we initially based our analysis on a mean date of 1654. Clearly, the range of possible dating estimates for VD21 is so narrow that using the widest possible range (i.e. 1643 or 1665) would have only a minor impact on our estimates of evolutionary rate and divergence times. The MCMC was run for 100 million generations (250 million in the case of the Bayesian skygrid analyses), and statistical confidence was assessed using values of the 95% Highest Posterior Density (HPD). All these analyses resulted in very similar nucleotide substitution rates and times to common ancestry, with tight and strongly overlapping distributions (Table S3), indicating that these results are robust to evolutionarily model. As the estimates using the strict clock and constant population size had the narrowest posterior distribution and the highest posterior probability we used these to infer the Maximum Clade Credibility (MCC) tree, with support for individual nodes available as posterior probability values (Table S3). Overlapping estimates of both the nucleotide substitution rate and divergence times across the tree were also obtained when VD21 was excluded from the BEAST analysis (Table S3), further indicating that the clock-like-structure in these data was not simply due to our ancient sample VD21. Finally, because there is a 7% chance that the correct date for the VD21 sequence is in fact 1785-1793, the BEAST analysis was repeated using the median date of 1789 and the same substitution and evolutionary models as described above. This again resulted in substitution rates that overlapped with those observed with the 1654 date, although estimates for the tMRCA for VARV as a whole were necessarily pushed toward the present (Table S3). However, the similarity of other divergence times across the phylogeny, including between P-I and P-II, indicates that VD21 alone is only making a minor impact on estimates of substitution rate and divergence time.

Finally, we used this same Bayesian approach to help validate the age of VD21. To do this we treated the age of VD21 as any other parameter, such that it is assigned a prior, and where the posterior corresponds to its estimated age with associated uncertainty. We used a uniform prior bounded between 0 and 500, corresponding to the years 1977 (i.e. the date of the most recent sample) and 1477. We used both constant size and Bayesian skygrid demographic models and a strict molecular clock. We did not use the lognormal relaxed clock model as it had very poor mixing indicating that it is over-parameterized. To assess whether the estimates were driven by the prior we repeated the analyses without the sequence data, which is equivalent to sampling from the prior distribution. If the sequence data are informative about the age of VD21, we expect that the prior and posterior will differ substantially. Importantly, both demographic models produced posterior estimates of the age of VD21 that differed considerably from the prior, and for which the mean estimates were close to the sampling year of VD21 (1654), at 1691 and 1665 for the constant size and the Bayesian skygrid models, respectively. In addition, the 95% credible intervals of both demographic models included the true sampling time: 1560–1882 for the constant size and 1553–1871 for the Bayesian skygrid.

Supplemental References

S1. Stuiver, M., and Polach, H.A. (1977). Reporting of C-14 data-Discussion. Radiocarbon *19*, 355-363.

S2. Shchelkunov, S.N., Blinov, V.M., and Sandakhchiev, L.S. (1993). Genes of variola and vaccinia viruses necessary to overcome the host protective mechanisms. FEBS letters *319*, 80-83.

S3. Shchelkunov, S.N., Totmenin, A.V., Loparev, V.N., Safronov, P.F., Gutorov, V.V., Chizhikov, V.E., Knight, J.C., Parsons, J.M., Massung, R.F., and Esposito, J.J. (2000). Alastrim smallpox variola minor virus genome DNA sequences. Virology *266*, 361-386.

S4. Esposito, J.J., Sammons, S.A., Frace, A.M., Osborne, J.D., Olsen-Rasmussen, M., Zhang, M., Govil, D., Damon, I.K., Kline, R., Laker, M., et al. (2006). Genome sequence diversity and clues to the evolution of variola (smallpox) virus. Science *313*, 807-812.

S5. Massung, R.F., Esposito, J.J., Liu, L.I., Qi, J., Utterback, T.R., Knight, J.C., Aubin, L., Yuran, T.E., Parsons, J.M., Loparev, V.N., et al. (1993). Potential virulence determinants in terminal regions of variola smallpox virus genome. Nature *366*, 748-751.

S6. Piombino-Mascali, D., and Jankauskas, R. (2014). Mummies of Lithuania. In Mummies around the World: an Encyclopedia of Mummies in History, Religion, and Popular Culture., M. Cardin, ed. (Santa Barbara: ABC-CLIO), pp. 246-247.

S7. Piombino-Mascali, D., Urbanavičius, A., Daubaras, M., Kozakaitė, J., Miliauskienė, Ž., and Jankauskas, R. (2015). The Lithuanian mummy project: A historical introduction. Lietuvos archeologija *41*, 131-142.

S8. Eidintas, A., Bumblauskas, A., Kulakauskas, A., Tamošaitis, M., and Vertimai, M. (2013). The history of Lithuania, (Eugrimas Publishing House).

S9. Urbanavičius, A. (2005). Vilniaus naujieji miestiečiai 1661-1795 m., (LII Leidykla).

S10. Dundulienė, P. (1963). Badas ir maras Lietuvoje feodalizmo laikais. Istorija *4*, 105-121.

S11. Karpiński, A. (2000). W walce z niewidzialnym wrogiem: epidemie chor′ob zaka′znych w Rzeczypospolitej w XVI-XVIII wieku i ich następstwa demograficzne, społeczno-ekonomiczne i polityczne, (Neriton).

S12. Scheuer, L., and Black, S. (2004). The Juvenile Skeleton, (Academic Press).

S13. Aufderheide, A.C. (2003). The scientific study of mummies, (Cambridge University Press).

S14. Beaumont, W., Beverly, R., Southon, J., and Taylor, R. (2010). Bone preparation at the KCCAMS laboratory. Nuclear Instruments and Methods in Physics Research Section B: Beam Interactions with Materials and Atoms *268*, 906-909.

S15. Reimer, P.J., Bard, E., Bayliss, A., Beck, J.W., Blackwell, P.G., Bronk Ramsey, C., Buck, C.E., Cheng, H., Edwards, R.L., and Friedrich, M. (2013). IntCal13 and Marine13 radiocarbon age calibration curves 0-50,000 years cal BP. Radiocarbon *55*, 1869-1887.

S16. Okello, J.B., Zurek, J., Devault, A.M., Kuch, M., Okwi, A.L., Sewankambo, N.K., Bimenya, G.S., Poinar, D., and Poinar, H.N. (2010). Comparison of methods in the recovery of nucleic acids from archival formalin-fixed paraffin-embedded autopsy tissues. Analytical Biochemistry *400*, 110-117.

S17. Dabney, J., Knapp, M., Glocke, I., Gansauge, M.-T., Weihmann, A., Nickel, B., Valdiosera, C., García, N., Pääbo, S., and Arsuaga, J.-L. (2013). Complete mitochondrial genome sequence of a Middle Pleistocene cave bear reconstructed from ultrashort DNA fragments. Proceedings of the National Academy of Sciences *110*, 15758-15763.

S18. Meyer, M., and Kircher, M. (2010). Illumina sequencing library preparation for highly multiplexed target capture and sequencing. Cold Spring Harbor Protocols doi:10.1101/pdb.prot5448.
S19. Kircher, M., Sawyer, S., and Meyer, M. (2012). Double indexing overcomes inaccuracies in multiplex sequencing on the Illumina platform. Nucleic Acids Research *40*, e3.
S20. Katoh, K., and Standley, D.M. (2013). MAFFT multiple sequence alignment software version 7: improvements in performance and usability. Molecular Biology and Evolution *30*, 772-780.
S21. Renaud, G., Stenzel, U., and Kelso, J. (2014). leeHom: adaptor trimming and merging for Illumina sequencing reads. Nucleic Acids Research *42*, e141.
S22. Li, H., and Durbin, R. (2009). Fast and accurate short read alignment with Burrows-Wheeler transform. Bioinformatics *25*, 1754-1760.
S23. Andrews, R.M., Kubacka, I., Chinnery, P.F., Lightowlers, R.N., Turnbull, D.M., and Howell, N. (1999). Reanalysis and revision of the Cambridge reference sequence for human mitochondrial DNA. Nature Genetics *23*, 147-147.
S24. Jónsson, H., Ginolhac, A., Schubert, M., Johnson, P.L., and Orlando, L. (2013). mapDamage2.0: fast approximate Bayesian estimates of ancient DNA damage parameters. Bioinformatics *29*, 1682-1684.
S25. Renaud, G., Slon, V., Duggan, A.T., and Kelso, J. (2015). Schmutzi: estimation of contamination and endogenous mitochondrial consensus calling for ancient DNA. Genome Biology *16*, 224.
S26. Weissensteiner, H., Pacher, D., Kloss-Brandstätter, A., Forer, L., Specht, G., Bandelt, H.-J., Kronenberg, F., Salas, A., and Schönherr, S. (2016). HaploGrep 2: mitochondrial haplogroup classification in the era of high-throughput sequencing. Nucleic Acids Research, gkw233.
S27. van Oven, M. (2015). PhyloTree Build 17: Growing the human mitochondrial DNA tree. Forensic Science International: Genetics Supplement Series *5*, e392-e394.
S28. Bankevich, A., Nurk, S., Antipov, D., Gurevich, A.A., Dvorkin, M., Kulikov, A.S., Lesin, V.M., Nikolenko, S.I., Pham, S., and Prjibelski, A.D. (2012). SPAdes: a new genome assembly algorithm and its applications to single-cell sequencing. Journal of Computational Biology *19*, 455-477.
S29. Gurevich, A., Saveliev, V., Vyahhi, N., and Tesler, G. (2013). QUAST: quality assessment tool for genome assemblies. Bioinformatics *29*, 1072-1075.
S30. Li, H. (2013). Aligning sequence reads, clone sequences and assembly contigs with BWA-MEM. arXiv preprint arXiv:1303.3997.
S31. Aguado, B., Selmes, I.P., and Smith, G.L. (1992). Nucleotide sequence of 21.8 kbp of variola major virus strain Harvey and comparison with vaccinia virus. Journal of General Virology *73*, 2887-2902.
S32. Alcami, A., and Smith, G.L. (1992). A soluble receptor for interleukin-1β encoded by vaccinia virus: a novel mechanism of virus modulation of the host response to infection. Cell *71*, 153-167.
S33. Moore, J.B., and Smith, G.L. (1992). Steroid hormone synthesis by a vaccinia enzyme: a new type of virus virulence factor. The EMBO Journal *11*, 1973-1980.
S34. Sonnhammer, E.L., and Durbin, R. (1995). A dot-matrix program with dynamic threshold control suited for genomic DNA and protein sequence analysis. Gene *167*, GC1-GC10.

S35. Sahl, J.W., Lemmer, D., Travis, J., Schupp, J., Gillece, J., Aziz, M., Driebe, E., Drees, K., Hicks, N., Williamson, C., et al. (2016). The Northern Arizona SNP Pipeline (NASP): accurate, flexible, and rapid identification of SNPs in WGS datasets. bioRxiv doi:http://dx.doi.org/10.1101/037267.

S36. McKenna, A., Hanna, M., Banks, E., Sivachenko, A., Cibulskis, K., Kernytsky, A., Garimella, K., Altshuler, D., Gabriel, S., Daly, M., et al. (2010). The Genome Analysis Toolkit: a MapReduce framework for analyzing next-generation DNA sequencing data. Genome Research *20*, 1297-1303.

S37. Talavera, G., and Castresana, J. (2007). Improvement of phylogenies after removing divergent and ambiguously aligned blocks from protein sequence alignments. Systematic Biology *56*, 564-577.

S38. Martin, D.P., Murrell, B., Golden, M., Khoosal, A., and Muhire, B. (2015). RDP4: Detection and analysis of recombination patterns in virus genomes. Virus Evolution *1*, vev003.

S39. Guindon, S., Dufayard, J.-F., Lefort, V., Anisimova, M., Hordijk, W., and Gascuel, O. (2010). New algorithms and methods to estimate maximum-likelihood phylogenies: assessing the performance of PhyML 3.0. Systematic Biology *59*, 307-321.

S40. Biagini, P., Thèves, C., Balaresque, P., Geraut, A., Cannet, C., Keyser, C., Nikolaeva, D., Gerard, P., Duchesne, S., and Orlando, L. (2012). Variola virus in a 300-year-old Siberian mummy. New England Journal of Medicine *367*, 2057-2059.

S41. Rambaut, A., Lam, T.T., Carvalho, L.M., and Pybus, O.G. (2016). Exploring the temporal structure of heterochronous sequences using TempEst (formerly Path-O-Gen). Virus Evolution *2*, vew007.

S42. Drummond, A.J., Suchard, M.A., Xie, D., and Rambaut, A. (2012). Bayesian phylogenetics with BEAUti and the BEAST 1.7. Molecular Biology and Evolution *29*, 1969-1973.
